# Basidiomycete non-reducing polyketide synthases function independently of SAT domains

**DOI:** 10.1186/s40694-023-00164-z

**Published:** 2023-08-04

**Authors:** Nikolai A. Löhr, Malik Rakhmanov, Jacob M. Wurlitzer, Gerald Lackner, Markus Gressler, Dirk Hoffmeister

**Affiliations:** 1 Institute of Pharmacy, Department Pharmaceutical Microbiology, Friedrich Schiller University Jena, Winzerlaer Strasse 2, 07745 Jena, Germany; 2 Department Pharmaceutical Microbiology, Leibniz Institute for Natural Product Research and Infection Biology – Hans Knöll Institute, Winzerlaer Strasse 2, 07745 Jena, Germany; 3 Synthetic Microbiology, Leibniz Institute for Natural Product Research and Infection Biology - Hans Knöll Institute, Winzerlaer Strasse 2, 07745 Jena, Germany

**Keywords:** Biosynthesis, Enzyme engineering, Orsellinic acid, Polyketide synthase, SAT domain

## Abstract

**Background:**

Non-reducing polyketide synthases (NR-PKSs) account for a major share of natural product diversity produced by both Asco- and Basidiomycota. The present evolutionary diversification into eleven clades further underscores the relevance of these multi-domain enzymes. Following current knowledge, NR-PKSs initiate polyketide assembly by an N-terminal starter unit:acyl transferase (SAT) domain that catalyzes the transfer of an acetyl starter from the acetyl-CoA thioester onto the acyl carrier protein (ACP).

**Results:**

A comprehensive phylogenetic analysis of NR-PKSs established a twelfth clade from which three representatives, enzymes CrPKS1-3 of the webcap mushroom *Cortinarius rufoolivaceus*, were biochemically characterized. These basidiomycete synthases lack a SAT domain yet are fully functional hepta- and octaketide synthases in vivo. Three members of the other clade of basidiomycete NR-PKSs (clade VIII) were produced as SAT-domainless versions and analyzed in vivo and in vitro. They retained full activity, thus corroborating the notion that the SAT domain is dispensable for many basidiomycete NR-PKSs. For comparison, the ascomycete octaketide synthase atrochrysone carboxylic acid synthase (ACAS) was produced as a SAT-domainless enzyme as well, but turned out completely inactive. However, a literature survey revealed that some NR-PKSs of ascomycetes carry mutations within the catalytic motif of the SAT domain. In these cases, the role of the domain and the origin of the formal acetate unit remains open.

**Conclusions:**

The role of SAT domains differs between asco- and basidiomycete NR-PKSs. For the latter, it is not part of the minimal set of NR-PKS domains and not required for function. This knowledge may help engineer compact NR-PKSs for more resource-efficient routes. From the genomic standpoint, seemingly incomplete or corrupted genes encoding SAT-domainless NR-PKSs should not automatically be dismissed as non-functional pseudogenes, but considered during genome analysis to decipher the potential arsenal of natural products of a given fungus.

**Supplementary Information:**

The online version contains supplementary material available at 10.1186/s40694-023-00164-z.

## Introduction

**Fig. 1:**
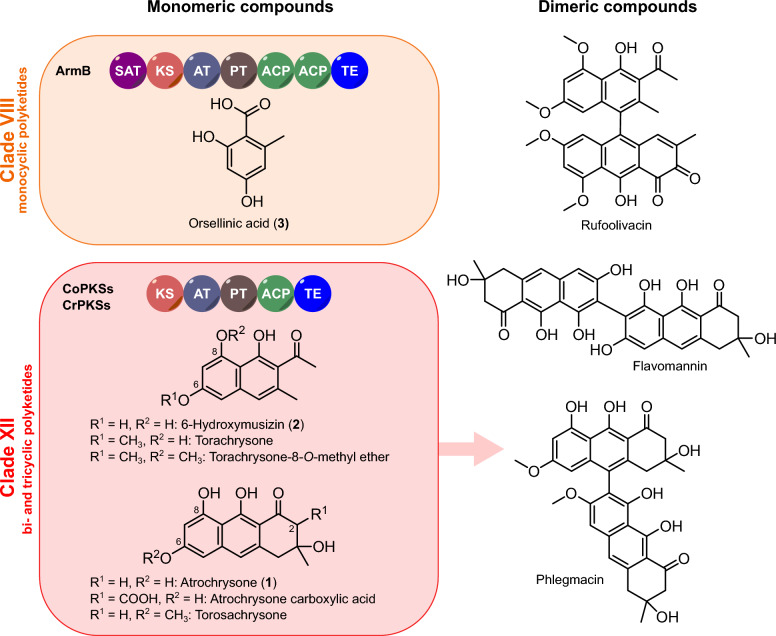
Chemical structures of basidiomycete monomeric and dimeric hepta- and octaketides. Clade VIII non-reducing polyketide synthases (NR-PKSs) comprise orsellinic acid (**3**) synthases, including ArmB [24] and others. Clade XII NR-PKSs are known from *Cortinarius* species [22], lack an N-terminal SAT domain and are associated with the synthesis of bi- and tricyclic polyketides such as 6-hydroxymusizin (**2**) and atrochrysone (**1**). In basidiomycetes, **1** and **2**, and their derivatives, often occur in dimeric form, as shown for phlegmacin (known from *C. odorifer* [36]), rufoolivacin (known from *C. rufoolivaceus* [36]) and flavomannin (known from *Tricholoma flavovirens* [40])

Polyketides are an intriguing class of bioactive fungal metabolites. They may impact our lives as hazardous mycotoxins that spoil food and contaminate feed, yet some also serve as invaluable pharmaceuticals [1]. Although mechanistically related to the rather uniform fatty acid biosynthesis [2], fungi have collectively evolved polyketide biosynthetic pathways to enable high structural diversity which simultaneously translates into a multitude of bioactivities. Despite this diversity, fungi rely almost exclusively on monomodular iterative Type I polyketide synthases (PKS) to produce polyketides. Enzymes of this class are composed of a set of domains that provide all catalytic activities to stepwise elongate and modify the nascent polyketide [3]. Various factors contribute to this structural diversity [4, 5]: (i) the choice of the starter unit (i.e., the first building block) that is extended by the PKS by stepwise condensation of malonyl-CoA derived acetate units, (ii) the number of extension cycles, which determine the chain length, (iii) in the case of reducing PKSs, the degree of reduction, (iv) the mode of offloading that may lead to linear or cyclic products, and (v) post-PKS modifications, such as dimerization [6], catalyzed by discrete tailoring enzymatic activities not integral to the PKS. For non-reducing (NR-)PKSs, starter unit selection is particularly critical and accomplished by the N-terminal starter unit:acyl transferase (SAT) domain unique to NR-PKSs [7]. The SAT domain typically selects acetyl-CoA which gives rise to the well-known starter unit effect [7–9]. However, some NR-PKSs use non-acetyl-CoA precursors, e.g., hexanoyl-CoA for aflatoxin [10], sterigmatocystin [11], and dothistromin [12] biosynthesis. Chain elongation occurs through the successive condensation of the growing polyketide with extender units, typically malonyl-CoA, which are supplied by the acyl transferase (AT) domain. The actual condensation reaction is then catalyzed by the ketosynthase (KS) domain [4, 5]. During the chain elongation process, the nascent polyketide is shuttled between the domains via a flexible phosphopantetheine arm of the acyl carrier protein (ACP). The product template (PT) domain ensures the correct cyclization of the highly reactive ACP-bound polyketide intermediates [13]. A variety of release mechanisms is known for fungal Type I PKS [4, 5, 14], e.g., hydrolytic or reductive release, cross-coupling, catalyzed by either a thioesterase (TE), Claisen-like cyclase (CLC), or a reductive release (R) domain. This set of domains entails an enzyme architecture that appears uniform yet turned out evolutionarily diverse: with increasing knowledge on fungal NR-PKSs and their phylogeny, the number of clades has increased from initially three to as many as eleven [15–20]. Interestingly, the members of all previously characterized clades, predominantly ascomycete enzymes, feature an N-terminal SAT domain. The minimal canonical domain set required for a functional NR-PKS was therefore considered SAT-KS-AT-PT-ACP. In addition, C-terminal TE/CLC/R domains that act either in *cis* or in *trans* release the polyketide product from the ACP [17]. A first intimation that basidiomycete PKSs can deviate from the standard domain setup came from an unnamed *Coprinopsis cinerea* PKS (the product of gene CC1G_05377 [21]) which lacks a SAT domain, even though closely related PKSs that fall into the same clade (clade VIII) possess a SAT domain. Recently, the paradigm of a SAT domain required for enzymatic activity has been questioned further as two basidiomycete NR-PKSs, CoPKS1 and CoPKS4 of *Cortinarius odorifer* (a Northern hemispheric ectomycorrhiza-forming webcap) feature the SAT-domainless architecture KS-AT-PT-ACP-TE [22, 23]. Yet, both enzymes were fully active in vivo and catalyzed the biosynthesis of atrochrysone (**1**) and 6-hydroxymusizin (**2**), i.e., tri- or bicyclic aromatic polyketides (Fig. 1). Moreover, in vitro assays demonstrated that malonyl-CoA serves as the exclusive source of acetate units and is sufficient for product formation [22]. In contrast, basidiomycete tetraketide synthases (clade VIII), e.g., the orsellinic acid synthase ArmB [24], possess a standard N-terminal SAT domain familiar from ascomycete NR-PKSs (Fig. 1). To resolve this discrepancy, we investigated a representative panel of asco- and basidiomycete NR-PKSs to determine whether the SAT domain was functionally required. To this end, we generated a series of truncated ΔSAT-PKSs and analyzed polyketide formation both in vitro and in *Aspergillus niger* as heterologous host. Here, we show that SAT domain-containing basidiomycete NR-PKSs remain active, even when the SAT domain is entirely removed. However, this does not seem to apply to the counterparts from ascomycetes, as a ΔSAT-variant of ACAS [25] from *Aspergillus terreus* did not yield any polyketide product compared to the full-length enzyme. Furthermore, we placed the genuinely SAT-domainless basidiomycete PKSs as a new and previously unrecognized twelfth clade in an evolutionary context with the established groups to provide a comprehensive phylogeny of NR-PKSs for the fungal kingdom.

## Results

### Phylogenetic placement of CoPKS1 and CoPKS4

**Fig. 2:**
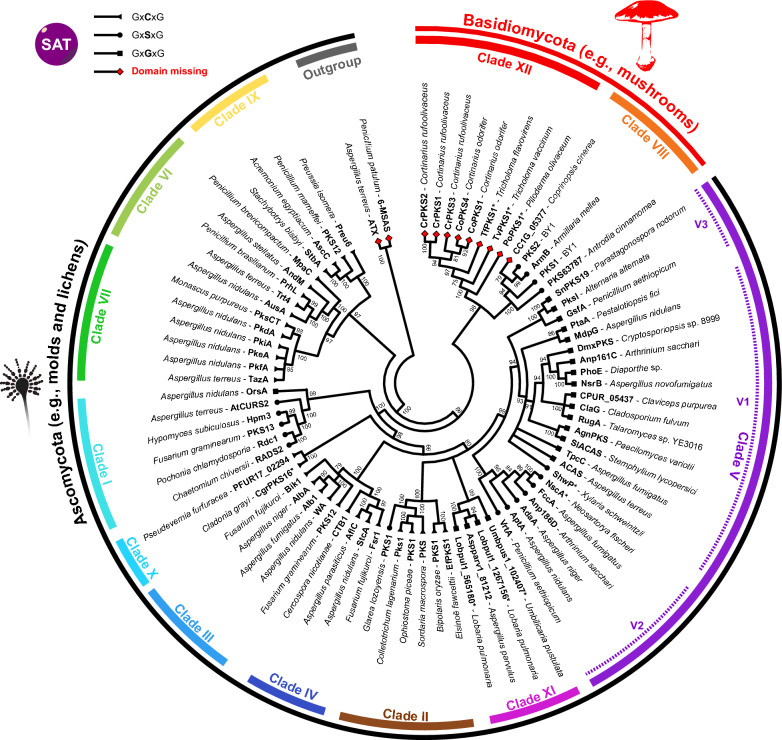
Phylogenetic reconstitution of fungal non-reducing polyketide synthases (NR-PKSs) based on the KS domains. A maximum likelihood phylogenetic tree was constructed using predominantly biochemically characterized NR-PKSs (Additional file 1: Table S7). NR-PKS from Ascomycota are highlighted by the outer black, basidiomycete NR-PKSs by the outer red arc. The inner arcs denote color-coded the twelve NR-PKS clades. Ultrafast bootstrap values of ≥ 75 are shown as node labels. The partially reducing PKS 6-MSAS of *Penicillium patulum* [97] and ATX of *Aspergillus terreus* [98] were used as outgroup. The symbols at the tips of the branches refer to the active site motifs of the respective SAT domains (triangle: Gx**C**xG /circle: Gx**S**xG/square: Gx**G**xG/red diamond: SAT-domain missing. Enzymes that have not yet been characterized biochemically in detail are highlighted with an asterisk (*)

Our previous work identified *Cortinarius* atrochrysone carboxylic acid synthases CoPKS1 and CoPKS4 as a distinct clade of NR-PKSs and clearly separated from previously characterized PKSs of identical activity [22]. To follow up on this finding, we determined the phylogenetic position of CoPKS1 and CoPKS4 in greater detail. Earlier reconstructions of the evolutionary history of fungal NR-PKSs followed different approaches and relied either on the complete PKS sequence, on the KS domain, or on the PT domain, yet resulted in congruent and comparable phylogenies [17, 26]. Here, we based our analyses on the ketosynthase as most conserved and most commonly used PKS domain to reconstruct NR-PKS evolution [27–29]. Maximum likelihood analysis unambiguously showed that the SAT-domainless NR-PKSs, i.e., the hepta- and octaketide synthases from basidiomycetes, form a distinct and robust phylogenetic clade within the KS tree (Fig. 2). Because of the close KS clustering, the distinct biochemical function (hepta- and octaketide formation) and the unique domain architecture (SAT-domainless), we suggest that this PKS family represents the twelfth characterized fungal PKS clade (clade XII). Interestingly, this clade shares a common ancestor with the basidiomycete tetraketide synthases (typically orsellinic acid synthases; clade VIII) but is phylogenetically distinct from the ascomycete hepta- or octaketide synthases (Fig. 2). The evolution of medium-chain polyketides from asco- and basidiomycetes can therefore be seen as a case of parallel evolution.

### Characterization of *Cortinarius rufoolivaceus* SAT-domainless NR-PKSs

**Fig. 3:**
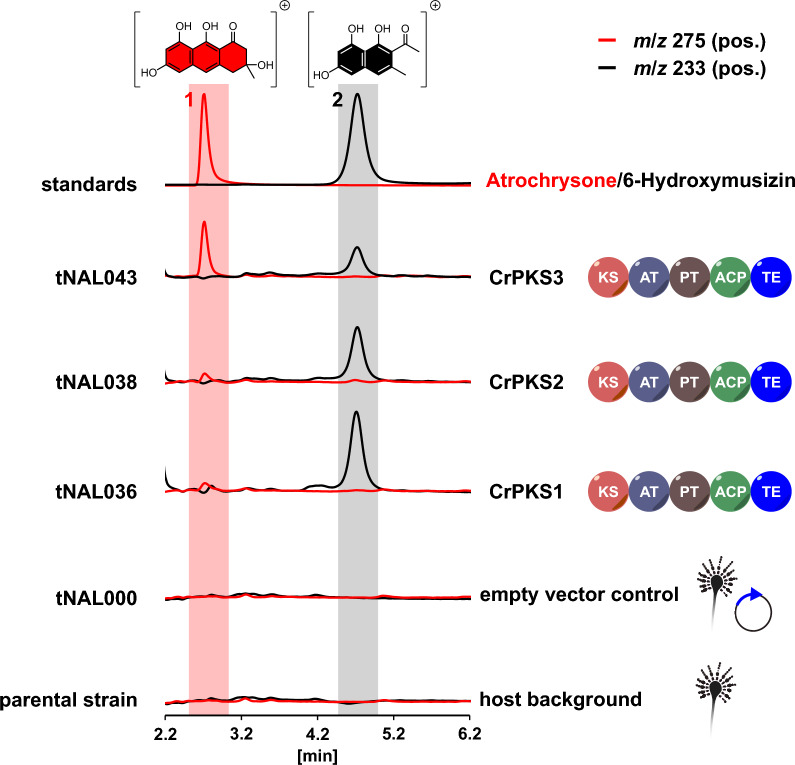
In vivo activity of *Cortinarius rufoolivaceus* NR-PKSs CrPKS1-CrPKS3 in *Aspergillus niger*. Overlaid extracted ion chromatograms (EICs) of ethyl acetate extracts are shown for *m*/*z* 275 [*M* + H]^+^ to detect the octaketide atrochrysone (**1,** red) and *m*/*z* 233 [*M* + H]^+^ for the heptaketide product 6-hydroxymusizin (**2**, black). Top panel: individual EICs for standards of **1** and **2**. Below: *A. niger* expressing the genes for CrPKS1 (*A. niger* tNAL036), CrPKS2 (tNAL038) and CrPKS3 (tNAL043). *A. niger* tNAL000 [22], carrying the insertless expression vector phis_SM-Xpress_URA, and the parental strain ATNT16Δ*pyrG*x24 were included as negative controls to monitor the metabolic background of the host

Initially, the clade XII was merely based on two characterized representatives, CoPKS1 and CoPKS4, taken from the same species, *C. odorifer*. To rule out that the SAT-domainless architecture of these particular *Cortinarius* PKSs reflects an exceptional singularity and to confirm they stand as generic representatives of a new clade of SAT-independent NR-PKSs, we investigated other representatives of clade XII. *Cortinarius rufoolivaceus* (the mottled webcap), like *C. odorifer*, is a confirmed producer of bi- and tricyclic aromatic polyketides [30] and a well investigated species with regard to natural product chemistry [31–33]. We sequenced the genome of the former and identified three candidate genes, encoding putative SAT-domainless NR-PKSs, now referred to as CrPKS1-CrPKS3. Subsequently, the genes were used to individually transform *Aspergillus niger* ATNT, designed for doxycycline-inducible transgene expression [34, 35]. After verifying the respective PKS genes had been fully integrated into the genome (Additional file 1: Fig. S1), the *A.* *niger* strains were cultured under conditions inducing transgene expression, and the ethyl acetate extracts of the culture broth were chromatographically analyzed. Heterologous expression of the genes encoding CrPKS1 (*A. niger* tNAL036), CrPKS2 (tNAL038), and CrPKS3 (tNAL043) led to fully active enzymes as evident from LC–MS analyses (Fig. 3). Consistent with previous findings for CoPKS1 and CoPKS4 [22], the *C. rufoolivaceus* enzymes CrPKS1-CrPKS3 catalyze simultaneous hepta- and octaketide formation. In contrast to their closely related *C. odorifer* PKS counterparts, the *C. rufoolivaceus* synthases CrPKS1 and CrPKS2 produce only low levels of the octaketide **1** and predominantly the heptaketide **2**. Intriguingly, the products found in the heterologous system reflected previous chemical analyses of *C. rufoolivaceus* fruiting bodies. These prior works had identified rufoolivacin, a heterodimer composed of an **1**-derived octaketide and a **2**-derived heptaketide, as their most prevalent natural product pigment (Fig. 1) [36]. In summary, our results on the activity of CrPKS1-CrPKS3 being hepta- and octaketide synthases are congruent with previous results on CoPKS1 and CoPKS4 which indicated that products of variable lengths are an intrinsic property of clade XII NR-PKSs. Moreover, our current results corroborate that NR-PKS activity does not require a SAT domain. Notably, members of clade XII PKSs are not restricted to the genus *Cortinarius* but occur more widespread in the division Basidiomycota. Through analyses of genomic data of mushrooms of other genera and phylogenetic origin, we found candidate genes encoding SAT-domainless NR-PKSs in *Piloderma olivaceum* (PoPKS1) [37], *Tricholoma vaccinum* (TvPKS1) [38] and *Tricholoma flavovirens* (TfPKS1) [39] (Additional file 1: Fig. S2), all of which phylogenetically cluster with CoPKSs and CrPKSs (Fig. 2). Furthermore, species of the genus *Tricholoma* are known producers of **1**-derived products, including flavomannin and congeners [40].

### SAT domains of other basidiomycete NR-PKSs

**Fig. 4:**
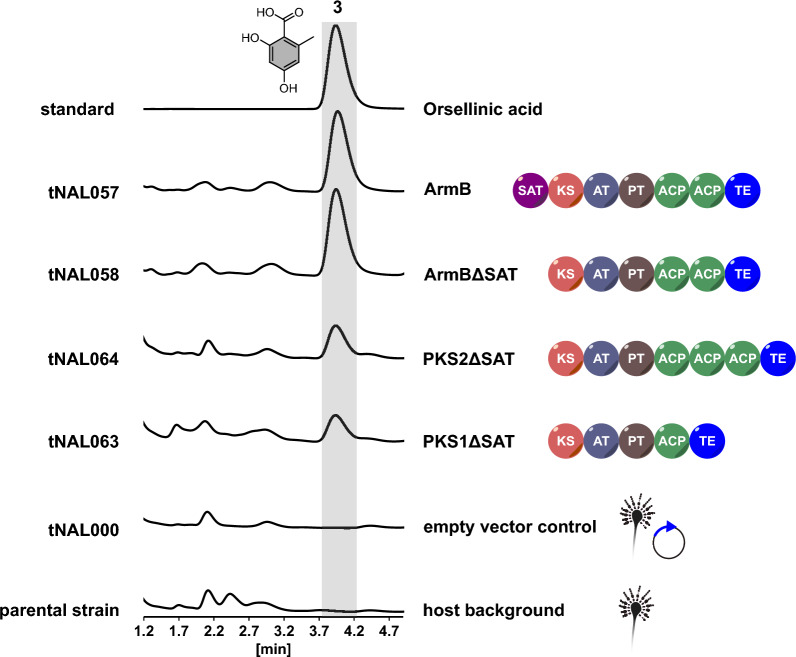
In vivo activity assays of ΔSAT-PKS of various mushrooms in *Aspergillus niger*. Chromatograms were recorded at *λ* = 254 nm. The top lane represents an authentic standard of orsellinic acid (**3**). Lower lanes: *A. niger* expressing the genes for full-length ArmB (*A. niger* tNAL057), ArmBΔSAT (tNAL058), PKS1ΔSAT (tNAL063), and PKS2ΔSAT (tNAL064). *A. niger* tNAL000 [22], containing the insertless expression vector phis_SM-Xpress_URA, and the parental strain ATNT16Δ*pyrG*x24 were included as negative controls to monitor the metabolic background of the host

Inspired by the SAT-domainless NR-PKS clade XII, we next surveyed the SAT domains in the other eleven fungal NR-PKS clades (Additional file 1: Fig. S3). We began with clade VIII, the second biochemically characterized clade from mushrooms (Fig. 2) which includes five biochemically characterized synthases catalyzing orsellinic acid (**3**) biosynthesis: ArmB from the honey mushroom *Armillaria mellea* [24], PKS1 and PKS2 from a taxonomically undescribed stereaceous basidiomycete referred to as BY1 [41], PKS63787 from the Taiwanese medicinal mushroom *Antrodia cinnamomea* [42, 43], and an unnamed protein encoded by the *Coprinopsis cinerea* CC1G_05377 gene [21]. While the first four PKSs share the canonical domain setup and differ only in a variable number of ACP domains (SAT-KS-AT-PT-ACP_n_-TE), the *C. cinerea* gene CC1G_05377 does not encode an N-terminal SAT domain (KS-AT-PT-ACP-TE). Thus, the SAT-domainless NR-PKS setup occurs in distantly related genera in mushrooms and indicates that the ancestral KS domain of basidiomycetes may already have possessed the ability to perform non-condensing decarboxylation reactions to provide the acetate starter. We therefore hypothesized that the SAT domain may be dispensable in other basidiomycete NR-PKSs as well, even if the domain is still present. To test this hypothesis, we generated genes encoding truncated, i.e., SAT-domainless variants of the NR-PKS clade VIII enzymes ArmB (ArmBΔSAT; *A. niger* tNAL058), PKS1 (PKS1ΔSAT; tNAL063), and PKS2 (PKS2ΔSAT; tNAL064) and assayed them for in vivo product formation. The transformant *A. niger* tNAL057 producing the full-length wildtype ArmB enzyme was included as positive control. A transformant (tNAL000) [22] carrying the insertless expression vector phis_SM-Xpress_URA along with the parental *s*train *A. niger* ATNT16Δ*pyrG*x24 served as negative controls. Once transgene integration was verified by diagnostic PCR (Additional file 1: Fig. S1), positive strains were grown in shake cultures, the broths extracted with ethyl acetate and the crude extracts were analyzed by LC–MS (Fig. 4). Surprisingly, all ΔSAT-enzymes were fully functional and produced **3** in vivo. In contrast, **3** was absent in the negative controls. These results indicate that multiple clade VIII PKSs do not depend on a SAT domain for product formation. This finding may even apply to all members of the clade and reflect a general feature of mushroom NR-PKSs.

### Priming assays with clade VIII NR-PKSs

**Fig. 5:**
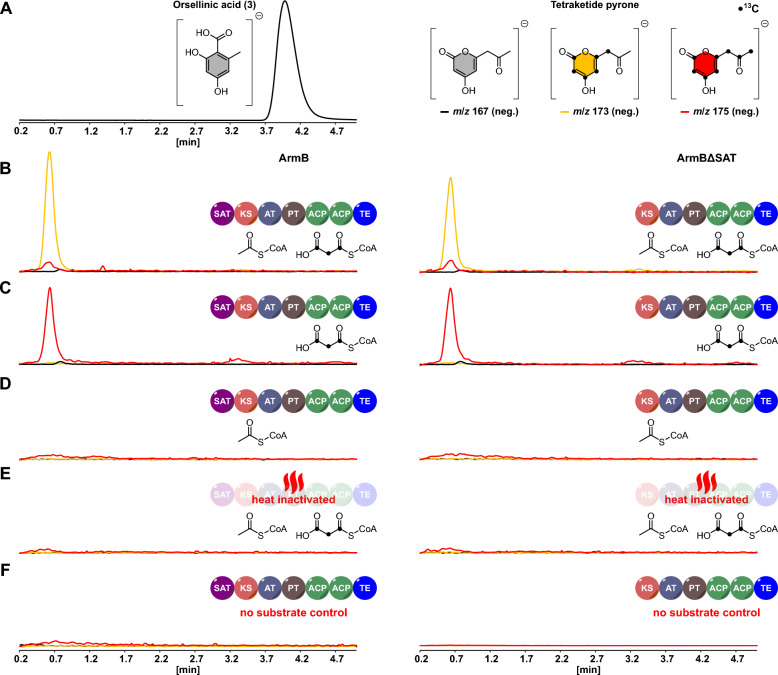
Chromatographic analysis of in vitro assays to test product formation by ArmB and ArmBΔSAT by stable-isotope labeling. The panels **B-F** show overlays of negative mode [*M *– H]^−^ extracted ion chromatograms (EICs) of the respective reactions and controls. EICs show *m*/*z* 167 (black lanes), *m*/*z* 173 (yellow lanes), and *m*/*z* 175 (red lanes) to detect tetraketides without, or with six or eight incorporated ^13^C atoms. **A** Authentic standard of orsellinic acid (**3**) and structures of the tetraketide pyrone, incorporated ^13^C atoms are indicated by black dots. **B-F** Analyses of the assays and control reactions. Left column: ArmB; right column: ArmBΔSAT. **B** Reactions with unlabeled acetyl-CoA and [^13^C_3_]malonyl-CoA. **C** Reactions with [^13^C_3_]malonyl-CoA as sole substrate. **D** Reactions with unlabeled acetyl-CoA only. **E** Negative controls with heat-inactivated enzymes and unlabeled acetyl-CoA and [^13^C_3_]malonyl-CoA substrates. **F** Negative controls, enzymes without substrates

**Fig. 6:**
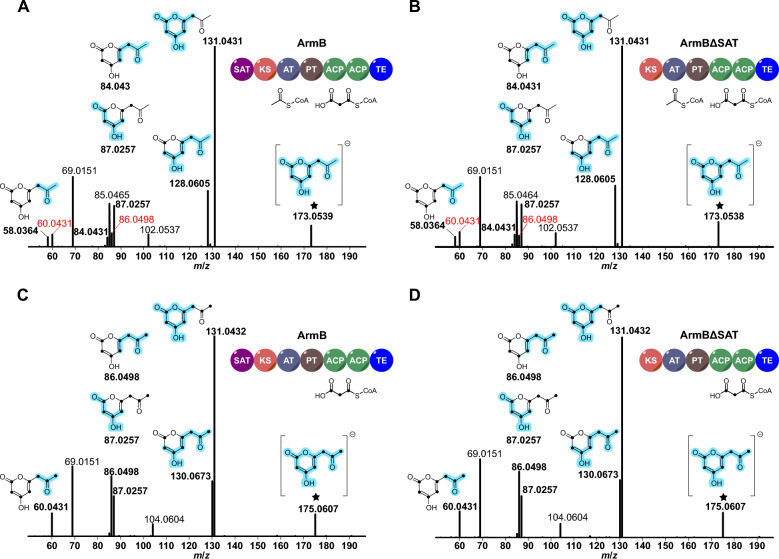
LC–MS/MS spectra of the tetraketide pyrone produced by ArmB and ArmBΔSAT in vitro. The spectra were recorded in negative [*M *– H]^−^ ionization mode. Asterisks denote the parental ions. Calculated masses for the tetraketide pyrone are *m*/*z* 173.0551 (^13^C_6_-labeled) and *m*/*z* 175.0607 (fully ^13^C_8_-labeled). Proposed fragments that are compatible with the highlighted mass values (bold) highlighted in light blue. **A** Analysis of the reactions with ArmB and unlabeled acetyl-CoA and [^13^C_3_]malonyl-CoA. **B** Reaction of ArmBΔSAT with unlabeled acetyl-CoA and [^13^C_3_]malonyl-CoA. **C** Reaction of ArmB with [^13^C_3_]malonyl-CoA only. **D** Reaction of ArmBΔSAT with [^13^C_3_]malonyl-CoA only. Mass signals in windows **A** and **B** which result from full labeling (.^13^C_8_) and which show high intensity in the spectra **C** and **D** are highlighted in red. A comprehensive comparison with literature data [46] on LC–MS/MS analyses of the tetraketide pyrone is provided in Additional file 1: Fig. S6

To exclude the possibility the truncated ΔSAT-enzymes were acetate-crossprimed, e.g., through a transfer from host fatty acid synthases [44], we subsequently analyzed ArmBΔSAT for product formation under in vitro conditions. Therefore, both ArmBΔSAT and the full-length ArmB were produced in *A. niger* tNAL058 and tNAL057, respectively, and purified as Strep-tag II fusion proteins [45] (Additional file 1: Fig. S4). Subsequently, the two enzymes were tested for product formation, using a substrate combination of unlabeled acetyl-CoA and stable-isotope labeled [^13^C_3_]malonyl-CoA. The reactions were analyzed by LC–MS in negative mode for expected mass traces of **3**. Curiously, both ArmB and ArmBΔSAT yielded a product eluting at a retention time (t_R_ = 0.6 min, Fig. 5) different from the authentic **3** standard (t_R_ = 3.9 min). As the detected masses (*m*/*z*) were in perfect agreement with those of **3**, we concluded that the product formed was the tetraketide pyrone (also known as tetraacetic acid lactone, TTL), which arises through O-C cyclization of the ACP-linked tetraketide [46] (Additional file 1: Fig. S5). Since pyrone formation can occur spontaneously [47, 48], PKSs occasionally display a different in vitro product spectrum in favor of pyrones, compared to in vivo reactions [49, 50]. To unequivocally confirm the identity of the TTL, subsequent LC–MS/MS analyses were carried out (Fig. 6), which were in perfect agreement with literature data [46, 51, 52] (Additional file 1: Fig. S6). Neither **3** nor TTL was present in negative controls, performed either with heat-inactivated proteins or without substrate (Fig. 5). The experimental setup also allowed for an analysis of the substrate preference of ArmB and ArmBΔSAT with respect to the incorporation of unlabeled acetyl-CoA and [^13^C_3_]malonyl-CoA. We thus monitored the extracted ion chromatograms (EICs) for *m*/*z* 167 [*M*-H]^−^ (mass for unlabeled **3**), *m*/*z* 173 (incorporation of six ^13^C atoms from three acetate units; ^12^C_2_^13^C_6_H_7_O_4_^−^) and *m*/*z* 175 (eight ^13^C atoms from all four acetate units; ^13^C_8_H_7_O_4_^−^). In reactions containing unlabeled acetyl-CoA and [^13^C_3_]malonyl-CoA, TTL was dominant in the EIC for *m*/*z* 173, but detectable in traces within the *m*/*z* 175 EIC as well. This result indicated the dominant PKS product was formed by incorporation one unit of acetyl-CoA and three units of malonyl-CoA. When [^13^C_3_]malonyl-CoA was added as sole substrate, TTL was detected exclusively in the *m*/*z* 175 EIC, indicating tetraketide formation from four malonyl-CoA units. High-resolution mass spectrometry showed partially or fully ^13^C-labeled species and confirmed these results (Fig. 6; calculated for ^12^C_2_^13^C_6_H_7_O_4_^−^ *m*/*z* 173.0551; detected *m*/*z* 173.0539 (calculated for ^13^C_8_H_7_O_4_^−^ *m*/*z* 175.0618; detected *m*/*z* 175.0607). As the product spectrum of ArmBΔSAT perfectly mirrored the one of full-length ArmB, we proved that ArmB is neither structurally nor catalytically dependent on the SAT domain. As previously shown for CoPKS4, malonyl-CoA is sufficient for product formation. Surprisingly, however, acetyl-CoA seems to be the preferred starter unit, even in the absence of the SAT domain. The observed in vitro formation of the TTL, although unusual, does not invalidate our conclusions about the use of starter units. Its synthesis may reflect a non-functional PT or TE domain caused by the extensive purification of large multi-domain enzymes (239 kDa and 199 kDa in size; Additional file 1: Fig. S4) [47].

Except *Antrodia cinnamomea* PKS63787 [42, 43], which was not included in our study, all biochemically characterized mushroom NR-PKSs either lack a SAT domain (clade XII enzymes CoPKS1, CoPKS4, CrPKS1, CrPKS2, CrPKS3 and *C. cinerea* clade VIII **3**-synthase) or were fully active when the SAT domain was artificially removed (clade VIII enzymes ArmB, PKS1, and PKS2). Thus, mushroom PKSs seem to not follow established knowledge about the minimal domain architecture of NR-PKSs and the starter unit effect [7]. From a mechanistic point of view, the SAT-independent enzymes from mushrooms appear evolutionarily most advanced as they synthesize (mono-, bi- and tricyclic) polyketides with the most minimalistic set of domains possible. In analogy to the starter unit effect, the term head start effect appears appropriate to describe the SAT domain-independent synthesis of polyketides by mushroom NR-PKSs. This phenomenon implies the AT domain loads all building blocks needed to initiate and extend the carbon chain. Furthermore, the KS domain likely catalyzes, in addition to the decarboxylative Claisen C–C-condensation reaction, a non-condensing decarboxylation of malonyl-CoA to provide the formal acetate starter. This is reminiscent of the bacterial Type II KS_β_ component [53] or the KS_Q_ of modular Type I PKSs [54].

### Analysis of SAT domains in ascomycete NR-PKSs

A biochemically characterized fully active NR-PKS lacking a SAT domain has not been reported yet for the Ascomycota. However, mutations within the catalytic motif of some SAT domains occur (Additional file 1: Fig. S3), most notably in clade V enzymes [25, 55, 56]. As this clade represents the most diverse class of NR-PKSs with numerous products and various cyclization patterns, it was further differentiated into three subclades (V1, V2 and V3; Additional file 1: Fig. S7) [26]. Subclade V1 includes the atrochrysone carboxylic acid synthases ACAS [25], MdpG [57], ClaG [58], among others, which represent the convergently evolved functional counterparts of the *Cortinarius* clade XII NR-PKSs. Mechanistically, loading of the starter unit requires thio- or oxoester chemistry involving either a cysteine or serine residue in the canonical Gx**C**xG/Gx**S**xG motif within the SAT domain [7, 8, 55]. Interestingly, a sequence analysis of subgroups V1-V3 revealed that the KS-based phylogeny (Additional file 1: Fig. S7) is congruent with the signature sequence of the SAT motif. While clade V2 enzymes share the canonical Gx**S**xG motif, almost all representatives of subgroups V1 and V3 feature a glycine at the corresponding position (Gx**G**xG) instead. Consistent with the apparently non-functional catalytic motif in clade V1 SAT domains, Watanabe et al. demonstrated that *Aspergillus terreus* ACAS [25] does not incorporate radioactively labeled [1-^14^C]acetyl-CoA and that malonyl-CoA as sole substrate is sufficient to produce **1**. This result suggests that the SAT domains of ACAS and possibly other clade V NR-PKSs may potentially also represent non-functional pseudodomains. However, a truncated SAT-domainless variant of ACAS has not been analyzed yet. We addressed this knowledge gap by creating genes encoding truncated variants of ACAS (ACASΔSAT), which we expressed heterologously in *A. niger* tNAL060 (*acas*Δ*sat* based on genomic DNA) and tNAL066 (*acas*Δ*sat* based on cDNA). As positive controls, the full-length variants of ACAS produced by *A. niger* tNAL059 (based on gDNA) and tNAL065 (cDNA) were added to our experiments. As ACAS depends on a discrete TE domain (ACTE) for product release, we based our in vivo assays on a double auxotrophic *A. niger* strain to co-express the genes for the TE domain. Genetic analyses by PCRs verified that the respective transgenes were properly integrated into the host genomes (Additional file 1: Fig. S8). After approximately 96 h of cultivation under inducing conditions, the strains expressing full-length ACASs turned intensely yellow, visually indicating polyketide production (Additional file 1: Fig. S9) while the cultures of strains producing the ΔSAT-variants and the negative control had not changed their color. Subsequently, the ethyl acetate extracts of all cultures were analyzed by LC–MS, which did not indicate either that polyketides had been produced by tNAL060 and tNAL066 (Additional file 1: Fig. S10). In contrast, in extracts from tNAL059 and tNAL065, the major metabolite was **1**, that is, the first stable product of ACAS-type synthases. We also detected the common set of minor follow-up compounds that are concomitantly formed upon heterologous production of ACAS-type enzymes in aspergilli, consisting of endocrocin and its anthrone dimers [22, 25, 57, 58].

We subsequently aimed at restoring PKS activity of the ΔSAT-producing strain tNAL066 by re-integrating the SAT domain as discrete stand-alone domain to interact *in trans* with the remaining ACAS tetradomain core (KS-AT-PT-ACP). Surprisingly, however, ACAS-activity was not restored in *A. niger* expression strain tNAL067 (Additional file 1: Fig. S9 and S10) after complementation of ACAS with a *trans-*acting SAT domain. We thus conclude that a SAT domain acting in *cis*, i.e., covalently attached to the other PKS domains, is important for the structural integrity of ACAS and hence indispensable for the overall activity, even if the domain may be catalytically inactive, as indicated by previous ^14^C-radionuclide assays [25].

## Discussion

In our study, a series of intrinsically SAT-domainless or artificially truncated basidiomycete NR-PKSs was functionally analyzed in vivo and in vitro. Our results suggest the SAT domain may be dispensable for basidiomycete PKS activity, yet parallel stable-isotope labeling assays with ArmB still identified acetyl-CoA as preferred starter. Crawford and Townsend explain the conservation of acetyl-CoA starter unit specificity by its immediate availability as citric acid cycle precursor [9]. Our observations confirm this interpretation as acetyl-CoA is still preferred over malonyl-CoA, even though with a SAT-domainless enzyme which is, however, flexible enough to use the latter for priming as well. We refer to this phenomenon and concept of SAT-independent synthesis of polyketides in mushrooms as head start effect. However, this concept does not seem valid for the ascomycete counterparts, as we demonstrated the SAT domain is essential, probably to maintain the structural integrity of the PKS. While most ascomycete SAT domains possess the canonical and catalytically competent active site motif Gx**S**xG or Gx**C**xG, the motif is changed to Gx**G**xG in many representatives of the ascomycete clade V NR-PKSs. Yet, despite this inert motif, the respective PKSs still remain active, as demonstrated by *A.* *terreus* ACAS [25]. Of note, ascomycete SAT domains may fulfill specialized functions beyond merely loading the starter unit. This is evident from the SAT domain of Preu6 from *Preussia isomera*, which is involved in the didepside formation of lecanoric acid [59]. Furthermore, SAT domains can act as gatekeeper for inter-enzymatic communication, known from an emerging class of characterized ascomycete NR-PKSs interacting with HR-PKSs in the biosynthesis of various bioactive compounds, such as radicicol [60, 61], zearalenone [62], asperfuranone [63], chaetoviridin [64, 65], hypothemicin [66, 67], sorbicillin [68, 69], or cladosporin [70]. In these examples, a HR-PKS, which lacks a release domain, synthesizes a polyketide starter which is transferred directly to the SAT domain of the partnering NR-PKS. However, inter-PKS communication was abolished in SAT^0^ domains in which the catalytically competent motifs were mutated [65, 67].

Such composite biosynthetic pathways are as yet unknown from basidiomycetes. This may reflect their propensity for rather short pathways that involve only a few catalytic steps [71] while aspergilli and other ascomycetes often rely on long multi-step pathways. Therefore, our results set the direction for future work, in particular pertaining to the chemical space accessible to and accessed by these fungi: while the SAT-domainless architecture of clade XII represents the most economical solution to produce di- or tricyclic natural product scaffolds, this domain setup precludes these pathways from evolving into mixed HR-/NR-PKS biosyntheses. Hence, cooperative pathways of fatty acid synthase-like enzymes with NR-PKSs, e.g., norsolorinic acid synthases AflC [72] and StcA [11, 73] of the aflatoxin and sterigmatocystin biosyntheses, cannot be established in a basidiomycete cell.

The question remains why the SAT domain was lost in clade XII synthases, while it has been preserved in most, but not all, synthases that belong to clade VIII that make **3**, i.e., a monocyclic product. From a mechanistic viewpoint, our results imply more far-reaching functionalities for the KS domains in clade XII NR-PKSs. Besides its role to condense the building blocks by establishing C–C bonds [4, 5] and its function to control the product chain length [23, 74], it must possess decarboxylating activity as well to provide the starter acetate units. The SAT-domainless architecture furthermore implies that the AT domain loads all building blocks required for both, carbon chain initiation and extension. Alternatively, the KS domain might even directly capture the starter unit to prime polyketide synthesis, as discussed earlier [75, 76]. Likewise, HR-PKSs also lack a SAT domain [77] but can nonetheless recruit unusual starter units, such as benzoate in strobilurin A [78] and squalestatin S1 [79] synthesis, or propionate in pseurotin A biosynthesis [80].

Besides the evolutionary and mechanistic perspective, our results have implications for biotechnology as well. SAT domains comprise several hundred amino acids. Synthetic biologists may appreciate new options to engineer more compact NR-PKSs for improved and resource-efficient routes toward anthraquinone precursors and other oligocyclic polyketides. Unlike their longer ascomycete counterparts, *Cortinarius* and other basidiomycete clade XII NR-PKSs offer both minimal sequence length and all functions integrated into one enzyme which eliminates the need of extra constructs to provide the discrete thioesterase domain.

## Conclusions

We conclude that the SAT domain is not part of the standard set of basidiomycete NR-PKS domains and not necessarily required for function. In the post-genomic era that generates an avalanche of genomic data [81], NR-PKS genes that do not encode SAT domains should not automatically be dismissed as non-functional pseudogenes. Rather, they deserve full attention by natural product researchers to uncover an even greater metabolic diversity produced by these remarkable fungal enzymes.

## Methods

### Organisms and culture conditions

Detailed information on fungal cultivation is provided in the additional experimental procedures in the supplementary information. All strains used in this study are listed in Additional file 1: Table S1.

### Genomic sequencing and cloning of *Cortinarius rufoolivaceus pks* genes

Genomic DNA of *Cortinarius rufoolivaceus* was sequenced using Oxford Nanopore Sequencing. To prepare a library for sequencing, 400 ng gDNA was processed with a rapid sequencing kit (Oxford Nanopore Technologies) according to the manufacturer’s instructions and sequenced on a MinION flow cell [82]. The genome was assembled as described earlier [83] using CANU v.1.9. [84, 85], based on an expected genome size of 60 Mbp. The biochemically characterized enzymes CoPKS1 and CoPKS4 [22] from the related webcap fungus *Cortinarius odorifer* were used as query to browse the (internally translated) genome of *C. rufoolivaceus* for similar sequences, using BlastP [86]. Models of the respective genes were predicted with Augustus [87]. Sequence analyses and alignments were carried out with Geneious software (version 7.1.9, Biomatters, Ltd.). To heterologously express *C. rufoolivaceus* polyketide synthases in *A. niger* ATNT16Δ*pyrG*x24, the intron-disrupted genes *crpks1*—*3* were amplified from gDNA using the oligonucleotides listed in Additional file 1: Table S2. The reaction contained 0.2 mM (each) deoxynucleoside triphosphate, 0.5 µM (each) oligonucleotide primer and 0.4 U Phusion high-fidelity DNA polymerase (NEB) in a total volume of 20 μL in HF buffer supplied with the enzyme. Primers were: oNAL186/187 to amplify *crpks1* and oNAL184/185 to amplify *crpks2* and *crpks3*. PCR parameters (condition I) are provided in Additional file 1: Table S3. All amplicons were electrophoretically purified on 0.7% (w/v) agarose gels and ligated to the *Nsi*I-linearized vector phis_SM-Xpress_URA [35, 88], using the NEBuilder HiFi DNA Assembly Master Mix (NEB). The created plasmids (Additional file 1: Table S4) were pNAL034 (to produce CrPKS1), pNAL035 (CrPKS2) and pNAL041 (CrPKS3). The integrity of the expression plasmids was verified by Sanger sequencing. GenBank accession numbers are: OQ863313 for *crpks1*, OQ863314 for *crpks2*, and OQ863315 for *crpks3*.

### Construction of expression plasmids for other native and SAT-domainless *pks* genes

To investigate the capacity of other NR-PKSs to synthesize polyketides in a SAT domain-independent manner, a series of plasmids (Additional file 1: Table S4) encoding truncated ΔSAT-NR-PKSs were generated. For all reactions, a total volume of 20 µL with 0.4 U of Phusion high-fidelity DNA polymerase (NEB) was used. The reaction mixtures contained 0.5 µM (each) oligonucleotide primer (Additional file 1: Table S2) and 0.2 mM (each) deoxynucleoside triphosphate in HF buffer supplied with the enzyme.

To produce Strep-tag II fusion proteins of both, a native and truncated ΔSAT-version of ArmB (lacking the first 381 aa), the respective cDNA-based gene fragments were amplified from pGL077 [24] using oNAL263/264 and oNAL266/267, respectively (condition II, Additional file 1: Table S3). The amplicons were electrophoretically purified on agarose gels and ligated into *Nsi*I-linearized pSM_StrepTag_X_URA [22] (to introduce a C-terminal Strep-tag II) using the NEBuilder HiFi DNA Assembly Master Mix (NEB) to yield pNAL053 (to produce full-length ArmB) and pNAL054 (ArmBΔSAT).

Plasmid pNAL061 to produce PKS1ΔSAT (lacking the first 389 aa) of the stereaceous basidiomycete BY1 and pNAL062 (PKS2ΔSAT; lacking the first 385 aa) were generated similarly, using primers oNAL278/279 to amplify the *pks1*Δ*sat* fragment from cDNA-based pJB117 [41] and primers oNAL281/282 to amplify the *pks2*Δ*sat* fragment from cDNA-based pJB115 [41] (condition I; Additional file 1: Table S3).

To generate plasmids encoding for ACAS and ACASΔSAT (lacking the first 268 aa) from *Aspergillus terreus* [25], the corresponding sequences were amplified from both gDNA and cDNA using pNAL055 and pNAL105 [23] as template, respectively. The intron-disrupted *acas*Δ*sat* gene was amplified with oNAL269/270 (condition I; Additional file 1: Table S3), electrophoretically purified on agarose gels and ligated into linearized *Nco*I-linearized pSM_StrepTag_X_URA using the NEBuilder HiFi DNA Assembly Master Mix (NEB) to yield pNAL056. The cDNA-based fragments were amplified (condition I; Additional file 1: Table S3) with oNAL322/269 (*acas*) and with oNAL321/269 (*acas*Δ*sat*) and ligated into *Nsi*I and *Nco*I opened phis_SM-Xpress to yield pNAL063 and pNAL064 using the NEBuilder HiFi DNA Assembly Master Mix (NEB). The plasmid encoding for the product-releasing ACTE thioesterase was generated using either the vector pSM_StrepTag_X_PABA [89] or phis_SM-Xpress_PABA (kindly provided by Dr. Matthias Brock, University of Nottingham, UK). Therefore, the *acte* gene was amplified (condition III; Additional file 1: Table S3) with oNAL274/oNAL323 from cDNA using pNAL106 [23] as template, purified in agarose gels, and assembled to pNAL065.

To produce a discrete SAT monodomain of ACAS, a vector was generated that is compatible with the ATNT system (i.e., transgene under control of P*terA*) and which contains a hygromycin resistance cassette (P*gpdA*:*hph*:T*trpC*). The *hph*-cassette was amplified from *hph*-pCRIV [90] using Phusion high-fidelity DNA polymerase (NEB) with oMG141/oMG138 and oMG137/oMG140 (condition IV; Additional file 1: Table S3) to introduce a silent mutation (GCC (Ala)—> GCA (Ala)) in the *hph*-gene, thereby removing an *Nco*I-restriction site. Subsequently, the two amplicons were joined via fusion-PCR (Additional file 1: Table S5), cut with *Not*I and ligated into *Not*I-opened SM-Xpress [34]. The resulting plasmid pMG04 thus contains an *hph*-cassette instead of the *ble*-resistance gene. The respective *acas* fragment encoding a SAT-monodomain was amplified from cDNA (pNAL105) [23] with oNAL319/320 (condition III; Additional file 1: Table S3) and ligated into *Nco*I-linearized pMG04 to yield pNAL066.

### Transformation of *Aspergillus niger*

For detailed information on protoplast transformation of *Aspergillus niger* and genetic analysis of transformants refer to the additional experimental procedures in the supplementary information.

### Heterologous production and purification of ArmB and ArmBΔSAT

To purify Strep-tag II fusion proteins of the full-length native ArmB and a SAT-domainless variant of ArmB (ArmBΔSAT) from *A. niger* tNAL057 and tNAL058, the Strep-Tactin Superflow resin and recommended buffers (iba life sciences) were used as previously described [22]. In brief, a 400 mL culture of yeast peptone dextrose medium (YPD), supplemented with 30 µg mL^−1^ doxycycline, was inoculated with 1 × 10^6^ conidia mL^−1^ and incubated for 36 h at 30 °C and 150 rpm. All subsequent steps for cell disruption and protein purification were performed as previously described [22]. Protein purity of the eluate fractions was verified by polyacrylamide gel electrophoresis (8% and 12% Laemmli gel, Additional file 1: Fig. S4). The eluates were repeatedly desalted on an Amicon Ultra-15 centrifugal filter unit (Merck, 100 kDa cutoff) and equilibrated in reaction buffer (see below). Protein concentrations were determined using the Pierce BCA Protein Assay Kit (Thermo Fisher) against a serial dilution of the supplied bovine serum albumin (BSA) standard.

### In vitro product formation assay

Product formation assays were carried out as duplicates in 250 µL reactions containing 0.5 μM purified protein in phosphate buffered saline (PBS, 137 mM NaCl, 2.7 mM KCl, 10 mM Na_2_HPO_4_, 1.8 mM KH_2_PO_4_, pH 7.4) with varying combinations of stable-isotope labeled acyl-CoA substrates. Substrates were: 2.5 mM unlabeled acetyl-CoA only (reaction condition I), 2.5 mM unlabeled acetyl-CoA plus 5 mM [^13^C_3_]malonyl-CoA (II), 5 mM [^13^C_3_]malonyl-CoA only (III) and a negative control lacking any substrate (IV). Reaction II was additionally performed with heat-inactivated enzymes (20 min, 95 °C) as negative controls. The reaction mixtures were incubated at 20 °C for 14 h. After lyophilization, the residue was dissolved in 50 µL MeOH and subjected to LC–MS (gradient II; Additional file 1: Table S6) to analyze product formation and stable-isotope incorporation.

### Analytical methods

For detailed information on liquid chromatography and mass spectrometry, refer to the additional experimental procedures in the supplementary information (Additional file 1: Table S6).

### Phylogenetic tree construction

Evolutionary analyses were based on the sequences of KS domains of biochemically characterized basidiomycete or ascomycete NR-PKSs, which are listed in Additional file 1: Table S7. Domains were predicted and annotated using the PFAM database [91]. The amino acid sequences of the respective KS domains were extracted and aligned using the MUSCLE algorithm [92] with 30,000 iterations. Improperly aligned regions were removed manually. Maximum likelihood trees were created using W-IQ-TREE [93] with Model Finder [94] and ultrafast bootstrapping [95]. Phylogenetic trees were visualized using MEGA X [96] and the cutoff value to condense branches was set to ≤ 75.

## Supplementary Information

**Additional file 1:** Additional experimental procedures, seven supplementary tables, and ten additional figures with supporting research data.

## Data Availability

The sequences of *crpks1-3* genes from *C. rufoolivaceus* are deposited under the GenBank accession numbers: OQ863313 for *crpks1*, OQ863314 for *crpks2*, and OQ863315 for *crpks3*.

## References

[1] Hoffmeister D, Keller NP (2007). Natural products of filamentous fungi: enzymes, genes, and their regulation. Nat Prod Rep.

[2] Smith S, Tsai SC (2007). The type I fatty acid and polyketide synthases: a tale of two megasynthases. Nat Prod Rep.

[3] Staunton J, Weissman KJ (2001). Polyketide biosynthesis: a millennium review. Nat Prod Rep.

[4] Hertweck C (2009). The biosynthetic logic of polyketide diversity. Angew Chem Int Ed.

[5] Cox RJ (2007). Polyketides, proteins and genes in fungi: programmed nano-machines begin to reveal their secrets. Org Biomol Chem.

[6] Hüttel W, Müller M (2021). Regio- and stereoselective intermolecular phenol coupling enzymes in secondary metabolite biosynthesis. Nat Prod Rep.

[7] Crawford JM, Dancy BCR, Hill EA, Udwary DW, Townsend CA (2006). Identification of a starter unit acyl-carrier protein transacylase domain in an iterative type I polyketide synthase. Proc Natl Acad Sci USA.

[8] Crawford JM, Vagstad AL, Whitworth KR, Ehrlich KC, Townsend CA (2008). Synthetic strategy of nonreducing iterative polyketide synthases and the origin of the classical “starter-unit effect”. Chem Bio Chem.

[9] Crawford JM, Townsend CA (2010). New insights into the formation of fungal aromatic polyketides. Nat Rev Microbiol.

[10] Townsend CA, Christensen SB, Trautwein K (1984). Hexanoate as a starter unit in polyketide biosynthesis. J Am Chem Soc.

[11] Yu JH, Leonard TJ (1995). Sterigmatocystin biosynthesis in *Aspergillus nidulans* requires a novel type I polyketide synthase. J Bacteriol.

[12] Bradshaw RE, Jin HP, Morgan BS, Schwelm A, Teddy OR, Young CA, Zhang SG (2006). A polyketide synthase gene required for biosynthesis of the aflatoxin-like toxin, dothistromin. Mycopathologia.

[13] Crawford JM, Korman TP, Labonte JW, Vagstad AL, Hill EA, Kamari-Bidkorpeh O, Tsai SC, Townsend CA (2009). Structural basis for biosynthetic programming of fungal aromatic polyketide cyclization. Nature.

[14] Du L, Lou L (2010). PKS and NRPS release mechanisms. Nat Prod Rep.

[15] Kroken S, Glass NL, Taylor JW, Yoder OC, Turgeon BG (2003). Phylogenomic analysis of type I polyketide synthase genes in pathogenic and saprobic ascomycetes. Proc Natl Acad Sci USA.

[16] Li Y, Xu W, Tang Y (2010). Classification, prediction, and verification of the regioselectivity of fungal polyketide synthase product template domains. J Biol Chem.

[17] Ahuja M, Chiang YM, Chang SL, Praseuth MB, Entwistle R, Sanchez JF, Lo HC, Yeh HH, Oakley BR, Wang CCC (2012). Illuminating the diversity of aromatic polyketide synthases in *Aspergillus nidulans*. J Am Chem Soc.

[18] Liu L, Zhang Z, Shao CL, Wang JL, Bai H, Wang CY (2015). Bioinformatical analysis of the sequences, structures and functions of fungal polyketide synthase product template domains. Sci Rep.

[19] Kim W, Liu R, Woo S, Kang KB, Park H, Yu YH, Ha HH, Oh SY, Yang JH, Kim H (2021). Linking a gene cluster to atranorin, a major cortical substance of lichens, through genetic dereplication and heterologous expression. MBio.

[20] Mosunova OV, Navarro-Munoz JC, Haksar D, van Neer J, Hoeksma J, den Hertog J, Collemare J (2022). Evolution-informed discovery of the naphthalenone biosynthetic pathway in fungi. MBio.

[21] Ishiuchi K, Nakazawa T, Ookuma T, Sugimoto S, Sato M, Tsunematsu Y, Ishikawa N, Noguchi H, Hotta K, Moriya H (2012). Establishing a new methodology for genome mining and biosynthesis of polyketides and peptides through yeast molecular genetics. Chem Bio Chem.

[22] Löhr NA, Eisen F, Thiele W, Platz L, Motter J, Hüttel W, Gressler M, Müller M, Hoffmeister D (2022). Unprecedented mushroom polyketide synthases produce the universal anthraquinone precursor. Angew Chem Int Ed.

[23] Löhr NA, Urban MC, Eisen F, Platz L, Hüttel W, Gressler M, Müller M, Hoffmeister D (2023). The ketosynthase domain controls chain length in mushroom oligocyclic polyketide synthases. Chem Bio Chem.

[24] Lackner G, Bohnert M, Wick J, Hoffmeister D (2013). Assembly of melleolide antibiotics involves a polyketide synthase with cross-coupling activity. Chem Biol.

[25] Awakawa T, Yokota K, Funa N, Doi F, Mori N, Watanabe H, Horinouchi S (2009). Physically discrete beta-lactamase-type thioesterase catalyzes product release in atrochrysone synthesis by iterative type I polyketide synthase. Chem Biol.

[26] Throckmorton K, Wiemann P, Keller NP (2015). Evolution of chemical diversity in a group of non-reduced polyketide gene clusters: using phylogenetics to inform the search for novel fungal natural products. Toxins.

[27] Keatinge-Clay AT (2012). The structures of type I polyketide synthases. Nat Prod Rep.

[28] Gontang EA, Gaudencio SP, Fenical W, Jensen PR (2010). Sequence-based analysis of secondary-metabolite biosynthesis in marine actinobacteria. Appl Environ Microbiol.

[29] Lackner G, Misiek M, Braesel J, Hoffmeister D (2012). Genome mining reveals the evolutionary origin and biosynthetic potential of basidiomycete polyketide synthases. Fungal Genet Biol.

[30] Gill M, Steglich W (1987). Pigments of fungi (Macromycetes). Fortschr Chem Org Nat.

[31] Zhang AL, Qin JC, Bai MS, Gao JM, Zhang YM, Yang SX, Laatsch H (2009). Rufoolivacin B, a novel polyketide pigment from the fruiting bodies of the fungus *Cortinarius rufo-olivaceus* (basidiomycetes). Chin Chem Lett.

[32] Thiele W, Froede R, Steglich W, Müller M (2020). Enzymatic formation of rufoschweinitzin, a binaphthalene from the basidiomycete *Cortinarius rufoolivaceus*. Chem Bio Chem.

[33] Gao JM, Qin JC, Pescitelli G, Di Pietro S, Ma YT, Zhang AL (2010). Structure and absolute configuration of toxic polyketide pigments from the fruiting bodies of the fungus *Cortinarius rufo-olivaceus*. Org Biomol Chem.

[34] Gressler M, Hortschansky P, Geib E, Brock M (2015). A new high-performance heterologous fungal expression system based on regulatory elements from the * Aspergillus terreus * terrein gene cluster. Front Microbiol.

[35] Geib E, Brock M (2017). ATNT: an enhanced system for expression of polycistronic secondary metabolite gene clusters in *Aspergillus niger*. Fungal Biol Biotechnol.

[36] Steglich W, Oertel B. Untersuchungen zur Konstitution und Verbreitung der Farbstoffe von *Cortinarius*, Untergattung *Phlegmacium* (*Agaricales*). Sydowia. 1984;37.

[37] Kohler A, Kuo A, Nagy LG, Morin E, Barry KW, Buscot F, Canbäck B, Choi C, Cichocki N, Clum A (2015). Convergent losses of decay mechanisms and rapid turnover of symbiosis genes in mycorrhizal mutualists. Nat Genet.

[38] Wagner K, Linde J, Krause K, Gube M, Koestler T, Sammer D, Kniemeyer O, Kothe E (2015). *Tricholoma vaccinum* host communication during ectomycorrhiza formation. FEMS Microbiol Ecol.

[39] Li H, Wu S, Ma X, Chen W, Zhang J, Duan S, Gao Y, Kui L, Huang W, Wu P (2018). The genome sequences of 90 mushrooms. Sci Rep.

[40] Steglich W, Töpfer-Petersen E, Reininger W, Gluchoff K, Arpin N (1972). Isolation of flavomannin-6,6′-dimethyl ether and one of its racemates from higher fungi. Phytochem.

[41] Braesel J, Fricke J, Schwenk D, Hoffmeister D (2017). Biochemical and genetic basis of orsellinic acid biosynthesis and prenylation in a stereaceous basidiomycete. Fungal Genet Biol.

[42] Yu PW, Chang YC, Liou RF, Lee TH, Tzean SS (2016). *Pks63787*, a polyketide synthase gene responsible for the biosynthesis of benzenoids in the medicinal mushroom *Antrodia cinnamomea*. J Nat Prod.

[43] Yu PW, Cho TY, Liou RF, Tzean SS, Lee TH (2017). Identification of the orsellinic acid synthase PKS63787 for the biosynthesis of antroquinonols in *Antrodia cinnamomea*. Appl Microbiol Biot.

[44] Foulke-Abel J, Townsend CA (2012). Demonstration of starter unit interprotein transfer from a fatty acid synthase to a multidomain, nonreducing polyketide synthase. Chem Bio Chem.

[45] Schmidt TGM, Skerra A (2007). The Strep-tag system for one-step purification and high-affinity detection or capturing of proteins. Nat Protoc.

[46] Feng Y, Yang X, Ji H, Deng Z, Lin S, Zheng J (2022). The *Streptomyces viridochromogenes* product template domain represents an evolutionary intermediate between dehydratase and aldol cyclase of type I polyketide synthases. Commun Biol.

[47] Kahlert L, Villanueva M, Cox RJ, Skellam EJ (2021). Biosynthesis of 6-hydroxymellein requires a collaborating polyketide synthase-like enzyme. Angew Chem Int Ed.

[48] Vagstad AL, Newman AG, Storm PA, Belecki K, Crawford JM, Townsend CA (2013). Combinatorial domain swaps provide insights into the rules of fungal polyketide synthase programming and the rational synthesis of non-native aromatic products. Angew Chem Int Ed.

[49] Reiter S, Cahn JKB, Wiebach V, Ueoka R, Piel J (2020). Characterization of an orphan type III polyketide synthase conserved in uncultivated “*Entotheonella*” sponge symbionts. Chem Bio Chem.

[50] Saxena P, Yadav G, Mohanty D, Gokhale RS (2003). A new family of type III polyketide synthases in *Mycobacterium tuberculosis*. J Biol Chem.

[51] Springob K, Samappito S, Jindaprasert A, Schmidt J, Page JE, De-Eknamkul W, Kutchan TM (2007). A polyketide synthase of *Plumbago indica* that catalyzes the formation of hexaketide pyrones. FEBS J.

[52] Taura F, Iijima M, Yamanaka E, Takahashi H, Kenmoku H, Saeki H, Morimoto S, Asakawa Y, Kurosaki F, Morita H (2016). A novel class of plant type III polyketide synthase involved in orsellinic acid biosynthesis from *Rhododendron dauricum*. Front Plant Sci.

[53] Hertweck C, Luzhetskyy A, Rebets Y, Bechthold A (2007). Type II polyketide synthases: gaining a deeper insight into enzymatic teamwork. Nat Prod Rep.

[54] Bisang C, Long PF, Cortes J, Westcott J, Crosby J, Matharu AL, Cox RJ, Simpson TJ, Staunton J, Leadlay PF (1999). A chain initiation factor common to both modular and aromatic polyketide synthases. Nature.

[55] Crawford JM, Vagstad AL, Ehrlich KC, Townsend CA (2008). Starter unit specificity directs genome mining of polyketide synthase pathways in fungi. Bioorg Chem.

[56] de Mattos-Shipley KMJ, Simpson TJ (2023). The 'emodin family' of fungal natural products-amalgamating a century of research with recent genomics-based advances. Nat Prod Rep.

[57] Chiang YM, Szewczyk E, Davidson AD, Entwistle R, Keller NP, Wang CCC, Oakley BR (2010). Characterization of the *Aspergillus nidulans* monodictyphenone gene cluster. Appl Environ Microb.

[58] Griffiths S, Mesarich CH, Saccomanno B, Vaisberg A, De Wit PJGM, Cox R, Collemare J (2016). Elucidation of cladofulvin biosynthesis reveals a cytochrome P450 monooxygenase required for anthraquinone dimerization. Proc Natl Acad Sci USA.

[59] Liu Q, Zhang D, Gao S, Cai X, Yao M, Xu Y, Gong Y, Zheng K, Mao Y, Yang L (2022). Didepside formation by the nonreducing polyketide synthase Preu6 of * Preussia isomera * requires interaction of starter acyl transferase and thioesterase domains. Angew Chem Int Ed.

[60] Wang S, Xu Y, Maine EA, Wijeratne EMK, Espinosa-Artiles P, Gunatilaka AAL, Molnár I (2008). Functional characterization of the biosynthesis of radicicol, an Hsp90 inhibitor resorcylic acid lactone from *Chaetomium chiversii*. Chem Biol.

[61] Zhou H, Qiao K, Gao Z, Vederas JC, Tang Y (2010). Insights into radicicol biosynthesis via heterologous synthesis ofintermediates and analogs. J Biol Chem.

[62] Zhou H, Zhan J, Watanabe K, Xie X, Tang Y (2008). A polyketide macrolactone synthase from the filamentous fungus *Gibberella zeae*. Proc Natl Acad Sci USA.

[63] Chiang YM, Szewczyk E, Davidson AD, Keller N, Oakley BR, Wang CCC (2009). A gene cluster containing two fungal polyketide synthases encodes the biosynthetic pathway for a polyketide, asperfuranone *Aspergillus nidulans*. J Am Chem Soc.

[64] Winter JM, Sato M, Sugimoto S, Chiou G, Garg NK, Tang Y, Watanabe K (2012). Identification and characterization of the chaetoviridin and chaetomugilin gene cluster in *Chaetomium globosum* reveal dual functions of an iterative highly-reducing polyketide synthase. J Am Chem Soc.

[65] Winter JM, Cascio D, Dietrich D, Sato M, Watanabe K, Sawaya MR, Vederas JC, Tang Y (2015). Biochemical and structural basis for controlling chemical modularity in fungal polyketide biosynthesis. J Am Chem Soc.

[66] Reeves CD, Hu Z, Reid R, Kealey JT (2008). Genes for the biosynthesis of the fungal polyketides hypothemycin from *Hypomyces subiculosus* and radicicol from *Pochonia chlamydosporia*. Appl Environ Microbiol.

[67] Zhou H, Qiao K, Gao Z, Meehan MJ, Li JW, Zhao X, Dorrestein PC, Vederas JC, Tang Y (2010). Enzymatic synthesis of resorcylic acid lactones by cooperation of fungal iterative polyketide synthases involved in hypothemycin biosynthesis. J Am Chem Soc.

[68] Guzmán-Chávez F, Salo O, Nygård Y, Lankhorst PP, Bovenberg RAL, Driessen AJM (2017). Mechanism and regulation of sorbicillin biosynthesis by *Penicillium chrysogenum*. Microb Biotechnol.

[69] Al Fahad A, Abood A, Fisch KM, Osipow A, Davison J, Avramović M, Butts CP, Piel J, Simpson TJ, Cox RJ (2014). Oxidative dearomatisation: the key step of sorbicillinoid biosynthesis. Chem Sci.

[70] Cochrane RVK, Sanichar R, Lambkin GR, Reiz B, Xu W, Tang Y, Vederas JC (2016). Production of new cladosporin analogues by reconstitution of the polyketide synthases responsible for the biosynthesis of this antimalarial agent. Angew Chem Int Ed.

[71] Gressler M, Löhr NA, Schäfer T, Lawrinowitz S, Seibold PS, Hoffmeister D (2021). Mind the mushroom: natural product biosynthetic genes and enzymes of basidiomycota. Nat Prod Rep.

[72] Yu J, Chang PK, Ehrlich KC, Cary JW, Bhatnagar D, Cleveland TE, Payne GA, Linz JE, Woloshuk CP, Bennett JW (2004). Clustered pathway genes in aflatoxin biosynthesis. Appl Environ Microbiol.

[73] Brown DW, Yu JH, Kelkar HS, Fernandes M, Nesbitt TC, Keller NP, Adams TH, Leonard TJ (1996). Twenty-five coregulated transcripts define a sterigmatocystin gene cluster in *Aspergillus nidulans*. Proc Natl Acad Sci USA.

[74] Liu T, Sanchez JF, Chiang YM, Oakley BR, Wang CCC (2014). Rational domain swaps reveal insights about chain length control by ketosynthase domains in fungal nonreducing polyketide synthases. Org Lett.

[75] Vagstad AL, Bumpus SB, Belecki K, Kelleher NL, Townsend CA (2012). Interrogation of global active site occupancy of a fungal iterative polyketide synthase reveals strategies for maintaining biosynthetic fidelity. J Am Chem Soc.

[76] Ma SM, Zhan J, Watanabe K, Xie X, Zhang W, Wang CC, Tang Y (2007). Enzymatic synthesis of aromatic polyketides using PKS4 from *Gibberella fujikuroi*. J Am Chem Soc.

[77] Cox RJ (2023). Curiouser and curiouser: progress in understanding the programming of iterative highly-reducing polyketide synthases. Nat Prod Rep.

[78] Nofiani R, de Mattos-Shipley K, Lebe KE, Han LC, Iqbal Z, Bailey AM, Willis CL, Simpson TJ, Cox RJ (2018). Strobilurin biosynthesis in basidiomycete fungi. Nat Commun.

[79] Bonsch B, Belt V, Bartel C, Duensing N, Koziol M, Lazarus CM, Bailey AM, Simpson TK, Cox RJ (2016). Identification of genes encoding squalestatin S1 biosynthesis and in vitro production of new squalestatin analogues. Chem Commun.

[80] Zou Y, Xu W, Tsunematsu Y, Tang M, Watanabe K, Tang Y (2014). Methylation-dependent acyl transfer between polyketide synthase and nonribosomal peptide synthetase modules in fungal natural product biosynthesis. Org Lett.

[81] Kenshole E, Herisse M, Michael M, Pidot SJ (2021). Natural product discovery through microbial genome mining. Curr Opin Chem Biol.

[82] Lu H, Giordano F, Ning Z (2016). Oxford Nanopore MinION sequencing and genome assembly. Genom Proteom Bioinform.

[83] Lawrinowitz S, Wurlitzer JM, Weiss D, Arndt HD, Kothe E, Gressler M, Hoffmeister D (2022). Blue light-dependent pre-mRNA splicing controls pigment biosynthesis in the mushroom *Terana caerulea*. Microbiol Spectr.

[84] Koren S, Walenz BP, Berlin K, Miller JR, Bergman NH, Phillippy AM (2017). Canu: scalable and accurate long-read assembly via adaptive k-mer weighting and repeat separation. Genome Res.

[85] Koren S, Rhie A, Walenz BP, Dilthey AT, Bickhart DM, Kingan SB, Hiendleder S, Williams JL, Smith TPL, Phillippy AM (2018). De novo assembly of haplotype-resolved genomes with trio binning. Nat Biotechnol.

[86] Altschul SF, Madden TL, Schäffer AA, Zhang J, Zhang Z, Miller W, Lipman DJ (1997). Gapped BLAST and PSI-BLAST: a new generation of protein database search programs. Nucleic Acids Res.

[87] Stanke M, Steinkamp R, Waack S, Morgenstern B (2004). AUGUSTUS: a web server for gene finding in eukaryotes. Nucleic Acids Res.

[88] Geib E, Baldeweg F, Doerfer M, Nett M, Brock M (2019). Cross-chemistry leads to product diversity from atromentin synthetases in Aspergilli from section Nigri. Cell Chem Biol..

[89] Wieder C, Peres da Silva R, Witts J, Jäger CM, Geib E, Brock M (2022). Characterisation of ascocorynin biosynthesis in the purple jellydisc fungus *Ascocoryne sarcoides*. Fungal Biol Biotechnol.

[90] Gressler M, Zaehle C, Scherlach K, Hertweck C, Brock M (2011). Multifactorial induction of an orphan PKS-NRPS gene cluster in *Aspergillus terreus*. Chem Biol.

[91] Mistry J, Chuguransky S, Williams L, Qureshi M, Salazar GA, Sonnhammer ELL, Tosatto SCE, Paladin L, Raj S, Richardson LJ (2021). Pfam: the protein families database in 2021. Nucleic Acids Res.

[92] Edgar RC (2004). MUSCLE: a multiple sequence alignment method with reduced time and space complexity. BMC Bioinform.

[93] Trifinopoulos J, Nguyen LT, von Haeseler A, Minh BQ (2016). W-IQ-TREE: a fast online phylogenetic tool for maximum likelihood analysis. Nucleic Acids Res.

[94] Kalyaanamoorthy S, Minh BQ, Wong TKF, von Haeseler A, Jermiin LS (2017). ModelFinder: fast model selection for accurate phylogenetic estimates. Nat Methods.

[95] Hoang DT, Chernomor O, von Haeseler A, Minh BQ, Vinh LS (2018). UFBoot2: improving the ultrafast bootstrap approximation. Mol Biol Evol.

[96] Kumar S, Stecher G, Li M, Knyaz C, Tamura K (2018). MEGA X: molecular evolutionary genetics analysis across computing platforms. Mol Biol Evol.

[97] Beck J, Ripka S, Siegner A, Schiltz E, Schweizer E (1990). The multifunctional 6-methylsalicylic acid synthase gene of *Penicillium patulum*. Eur J Biochem.

[98] Fujii I, Ono Y, Tada H, Gomi K, Ebizuka Y, Sankawa U (1996). Cloning of the polyketide synthase gene *atX* from *Aspergillus terreus* and its identification as the 6-methylsalicylic acid synthase gene by heterologous expression. Mol Gen Genet.

