# Supplementary material for: Basidiomycete non-reducing polyketide synthases function independently of SAT domains
Source: Fungal Biol Biotechnol. 2023 Aug 4;10:17. doi: 10.1186/s40694-023-00164-z (PMC10401856; doi:10.1186/s40694-023-00164-z)
Supplement: Supplementary file 1 — Additional file 1: Additional experimental procedures, seven supplementary tables, and ten additional figures with supporting research data. [file 40694_2023_164_MOESM1_ESM.pdf]

Basidiomycete non-reducing polyketide synthases function independently of SAT domains

Nikolai A. Löhr[1,2], Malik Rakhmanov[1,2], Jacob M. Wurlitzer[1,2], Gerald Lackner[3], Markus Gressler[1,2], Dirk Hoffmeister[1,2*]

[1] Institute of Pharmacy, Department Pharmaceutical Microbiology, Friedrich Schiller University Jena, Winzerlaer Strasse 2, 07745 Jena, Germany

[2] Department Pharmaceutical Microbiology, and

[3] Synthetic Microbiology, Leibniz Institute for Natural Product Research and Infection Biology − Hans Knöll Institute, Winzerlaer Strasse 2, 07745 Jena, Germany

[*]Correspondence: Dirk Hoffmeister, dirk.hoffmeister@leibniz-hki.de

Experimental Procedures

1. Microbiological methods

Strains and cultivation. For cloning and plasmid propagation, *Escherichia coli* XL1-Blue (Agilent) was routinely cultivated at 37 °C in lysogeny broth (LB) medium (10 g L^{-1} tryptone, 5 g L^{-1} yeast extract, and 10 g L^{-1} NaCl [pH 7.0]), supplemented with 100 µg mL^{-1} carbenicillin.

Cortinarius rufoolivaceus CBS 195.59 was maintained on solid malt extract-peptone (MEP) agar plates (30 g L^{-1} malt extract, 3 g L^{-1} soytone peptone, and 20 g L^{-1} agar [pH 5.6]) at 20 °C. For extraction and sequencing of chromosomal DNA, the fungus was grown in liquid MEP medium, shaken at 120 rpm, at 25 °C. The mycelium was collected after 26 d, rinsed with water, dried, and ground to a fine powder in liquid nitrogen. Isolation of genomic DNA (gDNA) followed a previously described protocol [1]. The gDNA was then precipitated with ice-cold isopropanol and washed several times with 70% ethanol. Eventually, the air-dried DNA pellet was dissolved in 10 mM Tris-HCl (pH 7.8) and prepared for nanopore sequencing.

The inducible *Aspergillus niger* ATNT system [2, 3] served as heterologous expression platform. Genotypes of individual transformants are listed in Table S1. All strains were routinely maintained at 30 °C on *Aspergillus* minimal medium (AMM) [4] supplemented with 100 mM D-glucose and 20 mM L-glutamine (AMM-G100Gln20). Conidia suspensions were harvested and quantified as described [5]. To induce transgene expression, doxycycline (30 µg mL^{-1}) was added to the cultures. For secondary metabolite analyses, liquid cultures of AMM-G100Gln20 (150 mL medium in 500 mL non-baffled stand flasks) were inoculated with 1×10^6 conidia mL^{-1} and incubated at 30 °C and 150 rpm, for 48 h.

To express two or three transgenes, the double auxotrophic strain ATNT16_2_No. 17.1 (Δ*pyrG*Δ*pabA*) was used, which was kindly provided by Dr. M. Brock, University of Nottingham, UK. For negative controls, the respective *A. niger* parental strains were used and supplemented with 10 mM uridine in case of ATNT16Δ*pyrG*x24 [3] and additionally with 7.3 µM *p*-aminobenzoic acid (paba) in case of the double auxotrophic strain ATNT16_2_No. 17.1 (Δ*pyrG*Δ*pabA*). *A. niger* tNAL000, harboring an insertless expression vector [5], served as additional control.

2. Molecular biology methods

Transformation of *A. niger* and genetic analysis of transformants. The transformation of *A. niger* protoplasts was carried out as previously described [6]. The selection of transformants was based on prototrophy on AMM-G100Gln20 agar plates, supplemented with 1.2 M sorbitol for osmotic stability. In case of tNAL067, transformants were additionally selected on plates containing 200 µg mL^{-1} hygromycin B. The isolation of chromosomal DNA of individual transformants was performed as previously described [1]. Diagnostic PCRs were carried out to verify the integration of the respective transgenes into the host genome using KOD DNA polymerase (Novagen) with supplied solutions according to the manufacturer's instructions (condition V, Table S2). For the single-transgene transformants (Fig. S1), oligonucleotides oMG360/oNAL156 were used. For strains containing more than one transgene, each was verified individually using oMG360 as forward primer and the respective reverse Gibson primer (Table S3), as shown in Fig. S8. For all transformants, three PCR-confirmed strains were used as replicates for metabolite analyses.

3. Analytical methods

Liquid chromatography and mass spectrometry. The culture broths of three independent strains per construct (three biological replicates) were analyzed independently. First, the broth was separated from the mycelium using miracloth and then extracted with equal volumes of ethyl acetate. The organic phase was collected, dried over anhydrous Na_2SO_4, evaporated to dryness, and dissolved in methanol (MeOH). The crude extracts were then subjected to UHPLC-MS analysis using a Nucleodur C18 Gravity column (guard column: 2.0 × 4 mm, 1.8 µm particle size; main column 50 mm × 2.0, 1.8 µm particle size; both thermostatted at 40 °C) on an Agilent 1290 Infinity II instrument, interfaced to an Agilent 6130 single quadrupole mass detector, which was operated in alternating positive/negative mode.

To analyze the crude extracts of *A. niger* strains tNAL036 (producing CrPKS1), tNAL038 (CrPKS2) and tNAL043 (CrPKS3) and strains producing variants of ACAS (tNAL059, tNAL060, tNAL065, tNAL066, tNAL067) gradient I was applied (Table S6). When required, extracted ion chromatograms (EICs) were recorded in the positive mode ($[M+H]^+$) to detect atrochrysone (**1**; *m/z* 275) and 6-hydroxymusizin (**2**; *m/z* 233), respectively. Authentic standards of **1** and **2** served as reference [5, 7]. To analyze the orsellinic acid (**3**) producing strains tNAL057, tNAL058, tNAL063 and tNAL064, gradient II was applied using the same instrument and column. An authentic synthetic standard of **3** served as reference.

HRESI-MS and tandem MS spectra were recorded on a Thermo Scientific Exactive Orbitrap instrument, using a reversed phase Accucore C18 column (100 × 2.1 mm, 2.6 µm) and applying gradient III (Table S6)

References

1. Girardin H, Latgé JP, Srikantha T, Morrow B, Soll DR. Development of DNA probes for fingerprinting *Aspergillus fumigatus*. J Clin Microbiol. 1993;31(6):1547-1554.
2. Geib E, Brock M. ATNT: an enhanced system for expression of polycistronic secondary metabolite gene clusters in *Aspergillus niger*. Fungal Biol Biotechnol. 2017;4:13.
3. Geib E, Baldeweg F, Doerfer M, Nett M, Brock M. Cross-chemistry leads to product diversity from atromentin synthetases in Aspergilli from section Nigri. Cell Chem Biol. 2019;26(2):223-234.
4. Pontecorvo G, Roper JA, Hemmons LM, Macdonald KD, Bufton AW. The genetics of *Aspergillus nidulans*. Adv Genet. 1953;5:141-238.
5. Löhr NA, Eisen F, Thiele W, Platz L, Motter J, Hüttel W, Gressler M, Müller M, Hoffmeister D. Unprecedented mushroom polyketide synthases produce the universal anthraquinone precursor. Angew Chem Int Ed. 2022;61(24):e202116142.
6. Geib E, Gressler M, Viediernikova I, Hillmann F, Jacobsen ID, Nietzsche S, Hertweck C, Brock M. A non-canonical melanin biosynthesis pathway protects *Aspergillus terreus* conidia from environmental stress. Cell Chem Biol. 2016;23(5):587-597.
7. Löhr NA, Urban MC, Eisen F, Platz L, Hüttel W, Gressler M, Müller M, Hoffmeister D. The ketosynthase domain controls chain length in mushroom oligocyclic polyketide synthases. ChemBioChem. 2023;24(3):e202200649.

Table S1 Fungal strains and their genotypes.

Strain	Genotype	Reference
Aspergillus niger ATNT16Δ*pyrG*x24	*TetOn*:*terR_ble*; Δ*pyrG*::*ptrA*	[1]
Aspergillus niger ATNT16_2_No. 17.1	*TetOn*:*terR_ble*; Δ*pyrG*::*ptrA*; Δ*pabA*	[2]
Aspergillus niger tNAL000	*TetOn*:*terR_ble*; Δ*pyrG*::*ptrA*; P*terA*:*His*$_6$*_pyrG*	[3]
Aspergillus niger tNAL036	*TetOn*:*terR_ble*; Δ*pyrG*::*ptrA*; P*terA*:*crpks1_pyrG*	This study
Aspergillus niger tNAL038	*TetOn*:*terR_ble*; Δ*pyrG*::*ptrA*; P*terA*:*crpks2_pyrG*	This study
Aspergillus niger tNAL043	*TetOn*:*terR_ble*; Δ*pyrG*::*ptrA*; P*terA*:*crpks3_pyrG*	This study
Aspergillus niger tNAL057	*TetOn*:*terR_ble*; Δ*pyrG*::*ptrA*; P*terA*:*armB_pyrG*	This study
Aspergillus niger tNAL058	*TetOn*:*terR_ble*; Δ*pyrG*::*ptrA*; P*terA*:*armB*Δ*sat_pyrG*	This study
Aspergillus niger tNAL059	*TetOn*:*terR_ble*; Δ*pyrG*::*ptrA*; Δ*pabA*; P*terA*:*acas_pyrG*; P*terA*:*acte_pabA*	[2]
Aspergillus niger tNAL060	*TetOn*:*terR_ble*; Δ*pyrG*::*ptrA*; Δ*pabA*; P*terA*:*acas*Δ*sat_pyrG*; P*terA*:*acte_pabA*	This study
Aspergillus niger tNAL063	*TetOn*:*terR_ble*; Δ*pyrG*::*ptrA*; P*terA*:*pks1*Δ*sat_pyrG*	This study
Aspergillus niger tNAL064	*TetOn*:*terR_ble*; Δ*pyrG*::*ptrA*; P*terA*:*pks2*Δ*sat_pyrG*	This study
Aspergillus niger tNAL065	*TetOn*:*terR_ble*; Δ*pyrG*::*ptrA*; Δ*pabA*; P*terA*:*acas_pyrG*; P*terA*:*acte_pabA*	This study
Aspergillus niger tNAL066	*TetOn*:*terR_ble*; Δ*pyrG*::*ptrA*; Δ*pabA*; P*terA*:*acas*Δ*sat _pyrG*; P*terA*:*acte_pabA*	This study
Aspergillus niger tNAL067	*TetOn*:*terR_ble*; Δ*pyrG*::*ptrA*; Δ*pabA*; P*terA*:*acas*Δ*sat _pyrG*; P*terA*:*acte_pabA*; P*terA*:*sat_hph*	This study
Cortinarius rufoolivaceus CBS 195.59	wild type	Westerdijk Institute

References

1. Geib E, Baldeweg F, Doerfer M, Nett M, Brock M. Cross-chemistry leads to product diversity from atromentin synthetases in Aspergilli from section Nigri. Cell Chem Biol. 2019;26(2):223-234.
2. Löhr NA, Urban MC, Eisen F, Platz L, Hüttel W, Gressler M, Müller M, Hoffmeister D. The ketosynthase domain controls chain length in mushroom oligocyclic polyketide synthases. ChemBioChem. 2023;24(3):e202200649.
3. Löhr NA, Eisen F, Thiele W, Platz L, Motter J, Hüttel W, Gressler M, Müller M, Hoffmeister D. Unprecedented mushroom polyketide synthases produce the universal anthraquinone precursor. Angew Chem Int Ed. 2022;61(24):e202116142.

Table S2 Oligonucleotides used in this study.

Name	Sequence (5' → 3')	Target	Purpose
oMG137	CGGTCGCGGAGGCAATGGATGCGATCGCTGC	*hph*-pCRIV	Mutation of *Nco*I site in *hph*-cassette
oMG138	GCAGCGATCGCATCCATTGCCTCCGCGACCG	*hph*-pCRIV	Mutation of *Nco*I site in *hph*-cassette
oMG140	GGTCGACTCTAGAGGATCCCCGCGGCCGCCAGTGTGATGGAATTCG	*hph*-pCRIV	Mutation of *Nco*I site in *hph*-cassette
oMG141	CGAATTCGAGCTCGGTACCCGCGGCCGCCAGTGTGATGGATATCTGC	*hph*-pCRIV	Mutation of *Nco*I site in *hph*-cassette
oMG360	CCTCCAAGAGAGATCCAGAC	P*terA*	Proof of transgene integration
oNAL156	GTGAGGGTTGAGTACGAGATT	T*trpC*	Proof of transgene integration
oNAL184	CTTGATCATTTAACAAACTTCTCATCACAGCACCATGCATCCACCAAATACTGCTAACAAAGCTGC	*crpks2/3*	Cloning of pNAL036 and pNAL041
oNAL185	CGAAACTATACGGTTCAGATTGAAATCACTGCTGTTATCCATGTCACCCCCTCTTATTCAACCAG	*crpks2/3*	Cloning of pNAL036 and pNAL041
oNAL186	CTTGATCATTTAACAAACTTCTCATCACAGCACCATGCATCCACCAAACACCGCTAACAAAGCTG	*crpks1*	Cloning of pNAL034
oNAL187	AACTATACGGTTCAGATTGAAATCACTGCTGTTATCCATGTCATCCCCGCTTGTTCAACCAGG	*crpks1*	Cloning of pNAL034
oNAL263	TCATTTAACAAACTTCTCATCACAGCACCATGCATTCGCCTTCTCTTGTGGTTCCTGTC	*armB*	Cloning of pNAL053
oNAL264	GCTGTTACCATGGCTTCTCGAACTGGGGGTGGGACCAAAGTTCATCCAGATACGCAC	*armB*	Cloning of pNAL053
oNAL266	CATTTAACAAACTTCTCATCACAGCACCATGCATACACTTCAGATGCCCTCGTCTG	*armB*	Cloning of pNAL054
oNAL267	GTTACCATGGCTTCTCGAACTGGGGGTGGGACCAAAGTTCATCCAGATACGCACATCC	*armB*	Cloning of pNAL054
oNAL269	TATACGGTTCAGATTGAAATCACTGCTGTTACCATGGGCTGTAATATTCTAGAAGCCAGC	*acas*	Cloning of pNAL056, pNAL063, pNAL064
oNAL270	ACAGCACCATGCATTGGTCCCACCCCCAGTTCGAGAAGCATGCAAAGCACATCTACACAG	*acas*	Cloning of pNAL056
oNAL274	TCATTTAACAAACTTCTCATCACAGCACCATGCATAAGCGGGGAGGCTACCGTCAAATC	*acte*	Cloning of pNAL065
oNAL278	TCTTGATCATTTAACAAACTTCTCATCACAGCACCATGCATGCTTCCTCTGAACCAAAT	*pks1*	Cloning of pNAL061
oNAL279	CTGCTGTTACCATGGCTTCTCGAACTGGGGGTGGGACCATAAGTCCAAATAATCACAGG	*pks1*	Cloning of pNAL061
oNAL281	TCTTGATCATTTAACAAACTTCTCATCACAGCACCATGCATCCTCCTCCTTCATCCTCG	*pks2*	Cloning of pNAL062
oNAL282	CTGCTGTTACCATGGCTTCTCGAACTGGGGGTGGGACCACAAAGACTCTAGGTAGACAC	*pks2*	Cloning of pNAL062
oNAL319	CTCTTGATCATTTAACAAACTTCTCATCACAGCACCATGGATTTCACCCCGTCGACAGG	*acas-sat*	Cloning of pNAL066
oNAL320	CCCGAAACTATACGGTTCAGATTGAAATCACTGCTGCTTAGCATAATCCACCGAATACG	*acas-sat*	Cloning of pNAL066
oNAL321	CTTGATCATTTAACAAACTTCTCATCACAGCACCATGCATGCAAAGCACATCTACACAG	*acas*	Cloning of pNAL064
oNAL322	TTGATCATTTAACAAACTTCTCATCACAGCACCATGCATGATTTCACCCCGTCGACAGG	*acas*	Cloning of pNAL063
oNAL323	GCCCGAAACTATACGGTTCAGATTGAAATCACTGCTGTTACGGGCTGGATGTGACATCA	*acte*	Cloning of pNAL065

Table S3 PCR parameters. Conditions I - IV were used for cloning using the Phusion high-fidelity DNA polymerase (NEB) and were initiated with denaturation for 30 s at 98 °C. Condition V was applied to diagnostic PCRs using the KOD DNA polymerase (Novagen). Here, the reactions were initiated with denaturation for 2 min at 94 °C. The elongation time for diagnostic PCRs was defined depending on the template and calculated according to the polymerase manufacturer's specifications of 1 min kb^{-1}. Expected amplicons are given in the Fig. S1 and Fig. S8.

Condition	Thermal cycling	Final elongation
I	35 cycles of 98 °C for 10 s, 60 °C for 15 s, 72 °C for 3 min	72 °C for 5 min
II	35 cycles of 98 °C for 10 s, 60 °C for 15 s, 72 °C for 4 min	72 °C for 8 min
III	35 cycles of 98 °C for 10 s, 60 °C for 15 s, 72 °C for 45 s	72 °C for 5 min
IV	30 cycles of 98 °C for 10 s, 60 °C for 15 s, 72 °C for 30 s	72 °C for 5 min
V	40 cycles of 98 °C for 10 s, 58 °C for 30 s, 68 °C for 1 min/kb	68 °C for 10 min

Table S4 Plasmids used in this study.

Plasmid name	Vector backbone	Gene	Reference
SM-Xpress	pUC19	-	[1]
phis_SM-Xpress_URA	pUC19	-	[2, 3]
pSM_StrepTag_X_URA	pUC19	-	[4]
phis_SM-Xpress_PABA	pUC19	-	unpublished
pSM_StrepTag_X_PABA	pUC19	-	[5, 6]
pGL077	pET28a	*armB* (cDNA)	[7]
pJB115	pET28b	*pks2* (cDNA)	[8]
pJB117	pET28b	*pks1* (cDNA)	[8]
pMG04	pUC19	-	This study
pNAL034	phis_SM-Xpress_URA	*crpks1* (gDNA)	This study
pNAL036	phis_SM-Xpress_URA	*crpks2* (gDNA)	This study
pNAL041	phis_SM-Xpress_URA	*crpks3* (gDNA)	This study
pNAL053	pSM_StrepTag_X_URA	*armB* (cDNA)	This study
pNAL054	pSM_StrepTag_X_URA	*armBΔsat* (cDNA)	This study
pNAL055	pSM_StrepTag_X_URA	*acas* (gDNA)	[6]
pNAL056	pSM_StrepTag_X_URA	*acasΔsat* (gDNA)	This study
pNAL057	pSM_StrepTag_X_PABA	*acte* (gDNA)	[6]
pNAL061	pSM_StrepTag_X_URA	*pks1Δsat* (cDNA)	This study
pNAL062	pSM_StrepTag_X_URA	*pks2Δsat* (cDNA)	This study
pNAL063	phis_SM-Xpress_URA	*acas* (cDNA)	This study
pNAL064	phis_SM-Xpress_URA	*acasΔsat* (cDNA)	This study
pNAL065	phis_SM-Xpress_PABA	*acte* (cDNA)	This study
pNAL066	pMG04	*sat* (cDNA)	This study
pNAL105	pJET1.2/blunt (CloneJET)	*acas* (cDNA)	[6]
pNAL106	pJET1.2/blunt (CloneJET)	*acte* (cDNA)	[6]

Table S5 Parameters for fusion-PCR.

	Thermal cycling	Final elongation
	7 cycles of 98 °C for 10 s, 60 °C for 15 s, 72 °C for 1 min	-
+ add primer	98 °C for 3 min	-
	7 cycles of 98 °C for 10 s, 60 °C for 15 s, 72 °C for 1 min	72 °C for 5 min

References

1. Gressler M, Hortschansky P, Geib E, Brock M. A new high-performance heterologous fungal expression system based on regulatory elements from the *Aspergillus terreus* terrein gene cluster. Front Microbiol. 2015;6:18.
2. Geib E, Brock M. ATNT: an enhanced system for expression of polycistronic secondary metabolite gene clusters in *Aspergillus niger*. Fungal Biol Biotechnol. 2017;4:13.
3. Geib E, Baldeweg F, Doerfer M, Nett M, Brock M. Cross-chemistry leads to product diversity from atromentin synthetases in Aspergilli from section Nigri. Cell Chem Biol. 2019;26(2):223-234.
4. Löhr NA, Eisen F, Thiele W, Platz L, Motter J, Hüttel W, Gressler M, Müller M, Hoffmeister D. Unprecedented mushroom polyketide synthases produce the universal anthraquinone precursor. Angew Chem Int Ed. 2022;61(24):e202116142.
5. Wieder C, Peres da Silva R, Witts J, Jäger CM, Geib E, Brock M. Characterisation of ascocorynin biosynthesis in the purple jellydisc fungus *Ascocoryne sarcoides*. Fungal Biol Biotechnol. 2022;9(1):8.
6. Löhr NA, Urban MC, Eisen F, Platz L, Hüttel W, Gressler M, Müller M, Hoffmeister D. The ketosynthase domain controls chain length in mushroom oligocyclic polyketide synthases. ChemBioChem. 2023;24(3):e202200649.
7. Lackner G, Bohnert M, Wick J, Hoffmeister D. Assembly of melleolide antibiotics involves a polyketide synthase with cross-coupling activity. Chem Biol. 2013;20(9):1101-1106.
8. Braesel J, Fricke J, Schwenk D, Hoffmeister D. Biochemical and genetic basis of orsellinic acid biosynthesis and prenylation in a stereaceous basidiomycete. Fungal Genet Biol. 2017;98:12-19.

Table S6 HPLC parameters. Eluents were: 0.1 % formic acid in water (eluent A) and acetonitrile (eluent B). In case of gradient III, eluent B was 0.1 % formic acid in acetonitrile.

Gradient	Flow [mL min^{-1}]	Time [min]	Eluent B [%]	Column
I	1	0.0	5	Nucleodur C18 Gravity (50 × 2.0 mm, 1.8 µm particle size)
		1.0	5	
		1.5	25	
	1.1	3.5	25	
		6.0	30	
	1	6.5	60	
		7.5	100	
II	1.0	0.0	5	Nucleodur C18 Gravity (50 × 2.0 mm, 1.8 µm particle size)
		6.5	5	
		7.5	100	
		8.5	100	
		9.5	5	
		10	5	
III	0.2	0.0	5	Accucore C18 column (100 × 2.1 mm, 2.6 µm particle size)
		10	98	
		22	99	

Table S7 Sequences of polyketide synthases used to reconstruct a phylogenetic tree. Enzymes highlighted in red are either partially characterized or deduced based on sequence similarity. The partially reducing PKS 6-MSAS from *Penicillium patulum* and ATX from *Aspergillus terreus* were used as outgroup (indicated as "OG").

Clade	Organism	Enzyme	Accession number	Reference
I	*Aspergillus nidulans*	**OrsA**	Q5AUX1.1	[1]
I	*Chaetomium chiversii*	**RADS2**	C5H882.1	[2]
I	*Pochonia chlamydosporia*	**Rdc1**	B3FWT6.1	[3]
I	*Fusarium graminearum*	**PKS13**	A0A098D6U0.1	[4]
I	*Hypomyces subiculosus*	**Hpm3**	B3FWS8.1	[5]
I	*Aspergillus terreus*	**AtCURS2**	L7XAV2.1	[6]
II	*Bipolaris oryzae*	**PKS1**	Q6L715	[7]
II	*Colletotrichum lagenarium*	**Pks1**	P79068	[8]
II	*Glarea lozoyensis*	**PKS1**	Q8J222	[9]
II	*Elsinoë fawcettii*	**EfPKS1**	A7UMW1.1	[10]
II	*Sordaria macrospora*	**PKS**	A7DWM3	[11]
II	*Ophiostoma piceae*	**PKS1**	EF125796.1	[12]
III	*Fusarium graminearum*	**PKS12**	I1RF58.1	[13]
III	*Fusarium fujikuroi*	**Bik1**	S0DZM7.1	[14]
III	*Aspergillus fumigatus*	**Alb1**	Q4WZA8.1	[15]
III	*Aspergillus nidulans*	**WA**	Q03149.2	[16]
III	*Aspergillus niger*	**AlbA**	A2QUI2.1	[17]
IV	*Aspergillus parasiticus*	**AflC**	Q12053.1	[18]
IV	*Cercospora nicotianae*	**CTB1**	Q6DQW3.1	[19]
IV	*Fusarium fujikuroi*	**Fsr1**	S0DTP6.1	[20]
IV	*Aspergillus nidulans*	**StcA**	Q12397.2	[21, 22]
V	*Aspergillus nidulans*	**AptA**	Q5B0D0.1	[23]
V	*Aspergillus nidulans*	**MdpG**	Q5BH30.1	[24]
V	*Aspergillus niger*	**AdaA**	G3KLH6.1	[25]
V	*Cladosporium fulvum*	**ClaG**	P0CU67.1	[26]
V	*Aspergillus terreus*	**ACAS**	Q0CCY3.1	[27]
V	*Stemphylium lycopersici*	**SlACAS**	KNG44542.1	[28]
V	*Aspergillus novofumigatus*	**NsrB**	A0A2I1C3X5.1	[29]
V	*Aspergillus fumigatus*	**TpcC**	Q4WQZ5.1	[30]

Table S7 (continued) Sequences of polyketide synthases used to reconstruct a phylogenetic tree. Enzymes highlighted in red are either partially characterized or deduced based on sequence similarity. The partially reducing PKS 6-MSAS from *Penicillium patulum* and ATX from *Aspergillus terreus* were used as outgroup (indicated as "OG").

Clade	Organism	Enzyme	Accession number	Reference
V	*Penicillium aethiopicum*	**GsfA**	D7PI15.1	[31]
V	*Penicillium aethiopicum*	**VrtA**	D7PHZ2.1	[31]
V	*Talaromyces* sp. YE3016	**RugA**	QZS37281.1	[32]
V	*Paecilomyces variotii*	**AgnPKS**	QBG38888.1	[33]
V	*Claviceps purpurea*	**CPUR_05437**	M1WG96.1	[34]
V	*Diaporthe* sp. SYSU-MS4722	**PhoE**	UPG58734.1	[35]
V	*Arthrinium sacchari*	**Anp161C**	None*	[36]
V	*Arthrinium sacchari*	**Anp186D**	None*	[36]
V	*Cryptosporiopsis* sp. 8999	**DmxPKS**	A0A4P8DJU2.1	[37]
V	*Xylaria schweinitzii*	**ShwP**	QLM00044.1	[38]
V	*Aspergillus fumigatus*	**FccA**	Q4WA61.1	[39, 40]
V	*Neosartorya fischeri*	**NscA**	A1D8I9.1	[40]
V	*Pestalotiopsis fici*	**PtaA**	A0A067XNI2.1	[41]
V	*Parastagonospora nodorum*	**SnPKS19**	A0A0H4ADX3.1	[42]
V	*Alternaria alternata*	**PksI**	RYN84545.1	[43] previously annotated differently [44]
VI	*Aspergillus nidulans*	**AusA**	Q5ATJ7.1	[45, 46]
VI	*Penicillium brevicompactum*	**MpaC**	F1DBA9.1	[47]
VI	*Aspergillus terreus*	**Trt4**	Q0C8A4.2	[48]
VI	*Aspergillus stellatus*	**AndM**	A0A097ZPE0.1	[49]
VI	*Penicillium brasilianum*	**PrhL**	A0A1E1FFN8.1	[50]
VII	*Aspergillus nidulans*	**PkdA**	Q5BG07.1	[51]
VII	*Aspergillus nidulans*	**PkeA**	Q5AUX7.2	[51]
VII	*Aspergillus nidulans*	**PkiA**	Q5B7U4.1	[51]
VII	*Aspergillus nidulans*	**PkfA**	Q5B8A0.1	[51]
VII	*Aspergillus terreus*	**TazA**	Q0CSA2.1	[52]
VII	*Monascus purpureus*	**PksCT**	Q65Z23.2	[53]
VIII	BY1**	**PKS1**	APH07629.1	[54]
VIII	BY1**	**PKS2**	APH07628.1	[54]
VIII	*Armillaria mellea*	**ArmB**	I3ZNU9.1	[55]
VIII	*Antrodia cinnamomea*	**PKS63787**	AST08390.1	[56]
VIII	*Coprinopsis cinerea*	**CC1G_05377**	XP_001835415.2	[57]

* Sequence not deposited with GenBank, but available in the literature reference.

** BY1 is a taxonomically unidentified stereaceous basidiomycete.

Table S7 (continued) Sequences of polyketide synthases used to reconstruct a phylogenetic tree. Enzymes highlighted in red are either partially characterized or deduced based on sequence similarity. The partially reducing PKS 6-MSAS from *Penicillium patulum* and ATX from *Aspergillus terreus* were used as outgroup (indicated as "OG").

Clade	Organism	Enzyme	Accession number	Reference
IX	*Stachybotrys bisbyi*	**StbA**	A0A193PS74.1	[58]
IX	*Penicillium marneffei*	**PKS12**	ADH01672.1	[59]
IX	*Acremonium egyptiacum*	**AscC**	A0A455R5P9.1	[60]
IX	*Preussia isomera*	**Preu6**	P9WET2.1	[61]
X	*Pseudevernia furfuracea*	**PFUR17_02294**	P9WES9.1	[62]
X	*Cladonia grayi*	**CgrPKS16**	ADM79459.1	[63]
XI	*Aspergillus parvulus*	**Aspparv1_81212**	None*	[64]
XI	*Lobaria pulmonaria*	**Lobpul1_1267156**	None*	[64]
XI	*Lobaria pulmonaria*	**Lobpul1_565180**	None*	[64]
XI	*Umbilicaria pustulata*	**Umbpus1_102407**	None*	[64]
XII	*Cortinarius odorifer*	**CoPKS1**	P9WES1	[65]
XII	*Cortinarius odorifer*	**CoPKS4**	P9WES2	[65]
XII	*Cortinarius rufoolivaceus*	**CrPKS1**	OQ863313	This study
XII	*Cortinarius rufoolivaceus*	**CrPKS2**	OQ863314	This study
XII	*Cortinarius rufoolivaceus*	**CrPKS3**	OQ863315	This study
XII	*Tricholoma flavovirens*	**TfPKS1**	None***	[66]
XII	*Tricholoma vaccinum*	**TvPKS1**	None***	[67]
XII	*Piloderma olivaceum*	**PoPKS1**	None***	[68]
OG	*Penicillium patulum*	**6-MSAS**	P22367.1	[69]
OG	*Aspergillus terreus*	**ATX**	Q0CJ59.1	[70]

* Sequence not deposited with GenBank, but available in the literature reference.

*** Sequence not separately deposited in GenBank but available through the published genome.

References

1. Gressler M, Hortschansky P, Geib E, Brock M. A new high-performance heterologous fungal expression system based on regulatory elements from the *Aspergillus terreus* terrein gene cluster. Front Microbiol. 2015;6:184.
2. Xu Y, Zhou T, Zhang S, Xuan L-J, Zhan J, Molnár I. Thioesterase domains of fungal nonreducing polyketide synthases act as decision gates during combinatorial biosynthesis. J Am Chem Soc. 2013;135(29):10783-10791.
3. Zhou H, Qiao K, Gao Z, Vederas JC, Tang Y. Insights into radicicol biosynthesis via heterologous synthesis of intermediates and analogs. J Biol Chem. 2010;285(53):41412-41421.
4. Wang M, Zhou H, Wirz M, Tang Y, Boddy CN. A thioesterase from an iterative fungal polyketide synthase shows macrocyclization and cross coupling activity and may play a role in controlling iterative cycling through product offloading. Biochem. 2009;48(27):6288-6290.
5. Reeves CD, Hu Z, Reid R, Kealey JT. Genes for the biosynthesis of the fungal polyketides hypothemycin from *Hypomyces subiculosus* and radicicol from *Pochonia chlamydosporia*. Appl Environ Microbiol. 2008;74(16):5121-5129.
6. Xu Y, Espinosa-Artiles P, Schubert V, Xu YM, Zhang W, Lin M, Gunatilaka AA, Süssmuth R, Molnár I. Characterization of the biosynthetic genes for 10,11-dehydrocurvularin, a heat shock response-modulating anticancer fungal polyketide from *Aspergillus terreus*. Appl Environ Microbiol. 2013;79(6):2038-2047.
7. Moriwaki A, Kihara J, Kobayashi T, Tokunaga T, Arase S, Honda Y. Insertional mutagenesis and characterization of a polyketide synthase gene (*PKS1*) required for melanin biosynthesis in *Bipolaris oryzae*. FEMS Microbiol Lett. 2004;238(1):1-8.
8. Vagstad AL, Hill EA, Labonte JW, Townsend CA. Characterization of a fungal thioesterase having Claisen cyclase and deacetylase activities in melanin biosynthesis. Chem Biol. 2012;19(12):1525-1534.
9. Zhang A, Lu P, Dahl-Roshak AM, Paress PS, Kennedy S, Tkacz JS, An Z. Efficient disruption of a polyketide synthase gene (*pks1*) required for melanin synthesis through *Agrobacterium*-mediated transformation of *Glarea lozoyensis*. Mol Genet Genom. 2003;268(5):645-655.
10. Liao HL, Chung KR. Genetic dissection defines the roles of elsinochrome phytotoxin for fungal pathogenesis and conidiation of the citrus pathogen *Elsinoe fawcettii*. Mol Plant Microbe Interact. 2008;21(4):469-479.
11. Engh I, Nowrousian M, Kück U. Regulation of melanin biosynthesis via the dihydroxynaphthalene pathway is dependent on sexual development in the ascomycete *Sordaria macrospora*. FEMS Microbiol Lett. 2007;275(1):62-70.
12. Tanguay P, Tangen K, Breuil C. Identifying pigmentation-related genes in *Ophiostoma piceae* using *Agrobacterium*-mediated integration. Phytopathology 2007;97(9):1040-1048.
13. Frandsen RJ, Schütt C, Lund BW, Staerk D, Nielsen J, Olsson S, Giese H. Two novel classes of enzymes are required for the biosynthesis of aurofusarin in *Fusarium graminearum*. J Biol Chem. 2011;286(12):10419-10428.
14. Linnemannstöns P, Schulte J, del Mar Prado M, Proctor RH, Avalos J, Tudzynski B. The polyketide synthase gene *pks4* from *Gibberella fujikuroi* encodes a key enzyme in the biosynthesis of the red pigment bikaverin. Fungal Genet Biol. 2002;37(2):134-148.
15. Tsai HF, Fujii I, Watanabe A, Wheeler MH, Chang YC, Yasuoka Y, Ebizuka Y, Kwon-Chung KJ. Pentaketide melanin biosynthesis in *Aspergillus fumigatus* requires chain-length shortening of a heptaketide precursor. J Biol Chem. 2001;276(31):29292-29298.
16. Fujii I, Watanabe A, Sankawa U, Ebizuka Y. Identification of Claisen cyclase domain in fungal polyketide synthase WA, a naphthopyrone synthase of *Aspergillus nidulans*. Chem Biol. 2001;8(2):189-197.
17. Chiang YM, Meyer KM, Praseuth M, Baker SE, Bruno KS, Wang CC. Characterization of a polyketide synthase in *Aspergillus niger* whose product is a precursor for both dihydroxynaphthalene (DHN) melanin and naphtho-gamma-pyrone. Fungal Genet Biol. 2011;48(4):430-437.
18. Yu J, Chang PK, Ehrlich KC, Cary JW, Bhatnagar D, Cleveland TE, Payne GA, Linz JE, Woloshuk CP, Bennett JW. Clustered pathway genes in aflatoxin biosynthesis. Appl Environ Microbiol. 2004;70(3):1253-1262.
19. Newman AG, Vagstad AL, Belecki K, Scheerer JR, Townsend CA. Analysis of the cercosporin polyketide synthase CTB1 reveals a new fungal thioesterase function. Chem Commun. 2012;48(96):11772-11774.
20. Studt L, Wiemann P, Kleigrewe K, Humpf HU, Tudzynski B. Biosynthesis of fusarubins accounts for pigmentation of *Fusarium fujikuroi* perithecia. Appl Environ Microbiol. 2012;78(12):4468-4480.
21. Yu JH, Leonard TJ. Sterigmatocystin biosynthesis in *Aspergillus nidulans* requires a novel type I polyketide synthase. J Bacteriol. 1995;177(16):4792-4800.
22. Brown DW, Yu JH, Kelkar HS, Fernandes M, Nesbitt TC, Keller NP, Adams TH, Leonard TJ. Twenty-five coregulated transcripts define a sterigmatocystin gene cluster in *Aspergillus nidulans*. Proc Natl Acad Sci USA. 1996;93(4):1418-1422.
23. Szewczyk E, Chiang YM, Oakley CE, Davidson AD, Wang CCC, Oakley BR. Identification and characterization of the asperthecin gene cluster of *Aspergillus nidulans*. Appl Environ Microb. 2008;74(24):7607-7612.

24. Chiang YM, Szewczyk E, Davidson AD, Entwistle R, Keller NP, Wang CCC, Oakley BR. Characterization of the *Aspergillus nidulans* monodictyphenone gene cluster. Appl Environ Microb. 2010;76(7):2067-2074.
25. Li YR, Chooi YH, Sheng YW, Valentine JS, Tang Y. Comparative characterization of fungal anthracenone and naphthacenedione biosynthetic pathways reveals an alpha-hydroxylation-dependent Claisen-like cyclization catalyzed by a dimanganese thioesterase. J Am Chem Soc. 2011;133(39):15773-15785.
26. Griffiths S, Mesarich CH, Saccomanno B, Vaisberg A, De Wit PJGM, Cox R, Collemare J. Elucidation of cladofulvin biosynthesis reveals a cytochrome P450 monooxygenase required for anthraquinone dimerization. Proc Natl Acad Sci USA. 2016;113(25):6851-6856.
27. Awakawa T, Yokota K, Funa N, Doi F, Mori N, Watanabe H, Horinouchi S. Physically discrete beta-lactamase-type thioesterase catalyzes product release in atrochrysone synthesis by iterative type I polyketide synthase. Chem Biol. 2009;16(6):613-623.
28. Sun L, Liu GY, Li Y, Jiang DY, Guo WF, Xu H, Zhan RT. Metabolic engineering of *Saccharomyces cerevisiae* for efficient production of endocrocin and emodin. Metab Eng. 2019;54:212-221.
29. Matsuda Y, Gotfredsen CH, Larsen TO. Genetic characterization of neosartorin biosynthesis provides insight into heterodimeric natural product generation. Org Lett. 2018;20(22):7197-7200.
30. Throckmorton K, Lim FY, Kontoyiannis DP, Zheng WF, Keller NP. Redundant synthesis of a conidial polyketide by two distinct secondary metabolite clusters in *Aspergillus fumigatus*. Environ Microbiol. 2016;18(1):246-259.
31. Chooi YH, Cacho R, Tang Y. Identification of the viridicatumtoxin and griseofulvin gene clusters from *Penicillium aethiopicum*. Chem Biol. 2010;17(5):483-494.
32. Han YB, Bai W, Ding CX, Liang J, Wu S-H, Tan RX. Intertwined biosynthesis of skyrin and rugulosin A underlies the formation of cage-structured bisanthraquinones. J Am Chem Soc. 2021;143(35):14218-14226.
33. Szwalbe AJ, Williams K, Song Z, de Mattos-Shipley K, Vincent JL, Bailey AM, Willis CL, Cox RJ, Simpson TJ. Characterisation of the biosynthetic pathway to agnestins A and B reveals the reductive route to chrysophanol in fungi. Chem Sci. 2019;10(1):233-238.
34. Neubauer L, Dopstadt J, Humpf HU, Tudzynski P. Identification and characterization of the ergochrome gene cluster in the plant pathogenic fungus *Claviceps purpurea*. Fungal Biol Biotechnol. 2016;3:2.
35. Yuan SW, Chen SH, Guo H, Chen LT, Shen HJ, Liu L, Gao ZZ. Elucidation of the complete biosynthetic pathway of phomoxanthone A and identification of a para–para selective phenol coupling dimerase. Org Lett. 2022;24(16):3069-3074.
36. Morishita Y, Okazaki Y, Luo YY, Nunoki J, Taniguchi T, Oshima Y, Asai T. Use of plant hormones to activate silent polyketide biosynthetic pathways in *Arthrinium sacchari*, a fungus isolated from a spider. Org Biomol Chem. 2019;17(4):780-784.
37. Greco C, de Mattos-Shipley K, Bailey AM, Mulholland NP, Vincent JL, Willis CL, Cox RJ, Simpson TJ. Structure revision of cryptosporioptides and determination of the genetic basis for dimeric xanthone biosynthesis in fungi. Chem Sci. 2019;10(10):2930-2939.
38. Thiele W, Froede R, Steglich W, Müller M. Enzymatic formation of rufoschweinitzin, a binaphthalene from the basidiomycete *Cortinarius rufoolivaceus*. ChemBioChem. 2020;21(10):1423-1427.
39. König CC, Scherlach K, Schroeckh V, Horn F, Nietzsche S, Brakhage AA, Hertweck C. Bacterium induces cryptic meroterpenoid pathway in the pathogenic fungus *Aspergillus fumigatus*. ChemBioChem. 2013;14(8):938-942.
40. Chooi YH, Fang J, Liu H, Filler SG, Wang P, Tang Y. Genome mining of a prenylated and immunosuppressive polyketide from pathogenic fungi. Org Lett. 2013;15(4):780-783.
41. Xu X, Liu L, Zhang F, Wang W, Li J, Guo L, Che Y, Liu G. Identification of the first diphenyl ether gene cluster for pestheic acid biosynthesis in plant endophyte *Pestalotiopsis fici*. ChemBioChem. 2014;15(2):284-292.
42. Chooi YH, Muria-Gonzalez MJ, Mead OL, Solomon PS. SnPKS19 encodes the polyketide synthase for alternariol mycotoxin biosynthesis in the wheat pathogen *Parastagonospora nodorum*. Appl Environ Microbiol. 2015;81(16):5309-5317.
43. Wenderoth M, Garganese F, Schmidt-Heydt M, Soukup ST, Ippolito A, Sanzani SM, Fischer R. Alternariol as virulence and colonization factor of *Alternaria alternata* during plant infection. Mol Microbiol. 2019;112(1):131-146.
44. Saha D, Fetzner R, Burkhardt B, Podlech J, Metzler M, Dang H, Lawrence C, Fischer R. Identification of a polyketide synthase required for alternariol (AOH) and alternariol-9-methyl ether (AME) formation in *Alternaria alternata*. PLoS One. 2012;7(7):e40564.
45. Nielsen ML, Nielsen JB, Rank C, Klejnstrup ML, Holm DK, Brogaard KH, Hansen BG, Frisvad JC, Larsen TO, Mortensen UH. A genome-wide polyketide synthase deletion library uncovers novel genetic links to polyketides and meroterpenoids in *Aspergillus nidulans*. FEMS Microbiol Lett. 2011;321(2):157-166.
46. Lo HC, Entwistle R, Guo CJ, Ahuja M, Szewczyk E, Hung JH, Chiang YM, Oakley BR, Wang CCC. Two separate gene clusters encode the biosynthetic pathway for the meroterpenoids austinol and dehydroaustinol in *Aspergillus nidulans*. J Am Chem Soc. 2012;134(10):4709-4720.

47. Regueira TB, Kildegaard KR, Hansen BG, Mortensen UH, Hertweck C, Nielsen J. Molecular basis for mycophenolic acid biosynthesis in *Penicillium brevicompactum*. Appl Environ Microb. 2011;77(9):3035-3043.
48. Itoh T, Tokunaga K, Radhakrishnan EK, Fujii I, Abe I, Ebizuka Y, Kushiro T. Identification of a key prenyltransferase involved in biosynthesis of the most abundant fungal meroterpenoids derived from 3,5-dimethylorsellinic acid. ChemBioChem. 2012;13(8):1132-1135.
49. Matsuda Y, Wakimoto T, Mori T, Awakawa T, Abe I. Complete biosynthetic pathway of anditomin: nature's sophisticated synthetic route to a complex fungal meroterpenoid. J Am Chem Soc. 2014;136(43):15326-15336.
50. Matsuda Y, Iwabuchi T, Fujimoto T, Awakawa T, Nakashima Y, Mori T, Zhang HP, Hayash F, Abe I. Discovery of key dioxygenases that diverged the paraherquonin and acetoxydehydroaustin pathways in *Penicillium brasilianum*. J Am Chem Soc. 2016;138(38):12671-12677.
51. Ahuja M, Chiang YM, Chang SL, Praseuth MB, Entwistle R, Sanchez JF, Lo HC, Yeh HH, Oakley BR, Wang CCC. Illuminating the diversity of aromatic polyketide synthases in *Aspergillus nidulans*. J Am Chem Soc. 2012;134(19):8212-8221.
52. Chiang YM, Oakley CE, Ahuja M, Entwistle R, Schultz A, Chang SL, Sung CT, Wang CCC, Oakley BR. An efficient system for heterologous expression of secondary metabolite genes in *Aspergillus nidulans*. J Am Chem Soc. 2013;135(20):7720-7731.
53. Shimizu T, Kinoshita H, Ishihara S, Sakai K, Nagai S, Nihira T. Polyketide synthase gene responsible for citrinin biosynthesis in *Monascus purpureus*. Appl Environ Microb. 2005;71(7):3453-3457.
54. Braesel J, Fricke J, Schwenk D, Hoffmeister D. Biochemical and genetic basis of orsellinic acid biosynthesis and prenylation in a stereaceous basidiomycete. Fungal Genet Biol. 2017;98:12-19.
55. Lackner G, Bohnert M, Wick J, Hoffmeister D. Assembly of melleolide antibiotics involves a polyketide synthase with cross-coupling activity. Chem Biol. 2013;20(9):1101-1106.
56. Yu PW, Chang YC, Liou RF, Lee TH, Tzean SS. *Pks63787*, a polyketide synthase gene responsible for the biosynthesis of benzenoids in the medicinal mushroom *Antrodia cinnamomea*. J Nat Prod. 2016;79(6):1485-1491.
57. Ishiuchi K, Nakazawa T, Ookuma T, Sugimoto S, Sato M, Tsunematsu Y, Ishikawa N, Noguchi H, Hotta K, Moriya H et al. Establishing a new methodology for genome mining and biosynthesis of polyketides and peptides through yeast molecular genetics. ChemBioChem. 2012;13(6):846-854.
58. Li C, Matsuda Y, Gao H, Hu D, Yao XS, Abe I. Biosynthesis of LL-Z1272 beta: discovery of a new member of NRPS-like enzymes for aryl-aldehyde formation. ChemBioChem. 2016;17(10):904-907.
59. Woo PCY, Lam CW, Tam EWT, Leung CKF, Wong SSY, Lau SKP, Yuen KY. First discovery of two polyketide synthase genes for mitorubrinic acid and mitorubrinol yellow pigment biosynthesis and implications in virulence of *Penicillium marneffei*. PLoS Negl Trop Dis. 2012;6(10).
60. Araki Y, Awakawa T, Matsuzaki M, Cho R, Matsuda Y, Hoshino S, Shinohara Y, Yamamoto M, Kido Y, Inaoka DK et al. Complete biosynthetic pathways of ascofuranone and ascochlorin in *Acremonium egyptiacum*. Proc Natl Acad Sci USA. 2019;116(17):8269-8274.
61. Liu QP, Zhang D, Gao SB, Cai XH, Yao M, Xu Y, Gong YF, Zheng K, Mao YG, Yang LY et al. Didepside formation by the nonreducing polyketide synthase Preu6 of *Preussia isomera* requires interaction of starter acyl transferase and thioesterase domains. Angew Chem Int Ed. 2022.
62. Kealey JT, Craig JP, Barr PJ. Identification of a lichen depside polyketide synthase gene by heterologous expression in *Saccharomyces cerevisiae*. Metab Eng Commun. 2021;13:e00172.
63. Armaleo D, Sun XM, Culberson C. Insights from the first putative biosynthetic gene cluster for a lichen depside and depsidone. Mycologia 2011;103(4):741-754.
64. Mosunova OV, Navarro-Muñoz JC, Haksar D, van Neer J, Hoeksma J, den Hertog J, Collemare J. Evolution-informed discovery of the naphthalenone biosynthetic pathway in fungi. MBio. 2022;13(3).
65. Löhr NA, Eisen F, Thiele W, Platz L, Motter J, Hüttel W, Gressler M, Müller M, Hoffmeister D. Unprecedented mushroom polyketide synthases produce the universal anthraquinone precursor. Angew Chem Int Ed. 2022;61(24):e202116142.
66. Li H, Wu S, Ma X, Chen W, Zhang J, Duan S, Gao Y, Kui L, Huang W, Wu P et al. The genome sequences of 90 mushrooms. Sci Rep. 2018;8(1):9982.
67. Wagner K, Linde J, Krause K, Gube M, Koestler T, Sammer D, Kniemeyer O, Kothe E. *Tricholoma vaccinum* host communication during ectomycorrhiza formation. FEMS Microbiol Ecol. 2015;91(11).
68. Kohler A, Kuo A, Nagy LG, Morin E, Barry KW, Buscot F, Canbäck B, Choi C, Cichocki N, Clum A et al. Convergent losses of decay mechanisms and rapid turnover of symbiosis genes in mycorrhizal mutualists. Nat Genet. 2015;47(4):410-415.
69. Beck J, Ripka S, Siegner A, Schiltz E, Schweizer E. The multifunctional 6-methylsalicylic acid synthase gene of *Penicillium patulum*. Eur J Biochem. 1990;192(2):487-498.
70. Fujii I, Ono Y, Tada H, Gomi K, Ebizuka Y, Sankawa U. Cloning of the polyketide synthase gene *atX* from *Aspergillus terreus* and its identification as the 6-methylsalicylic acid synthase gene by heterologous expression. Mol Gen Genet. 1996;253(1-2):1-10.

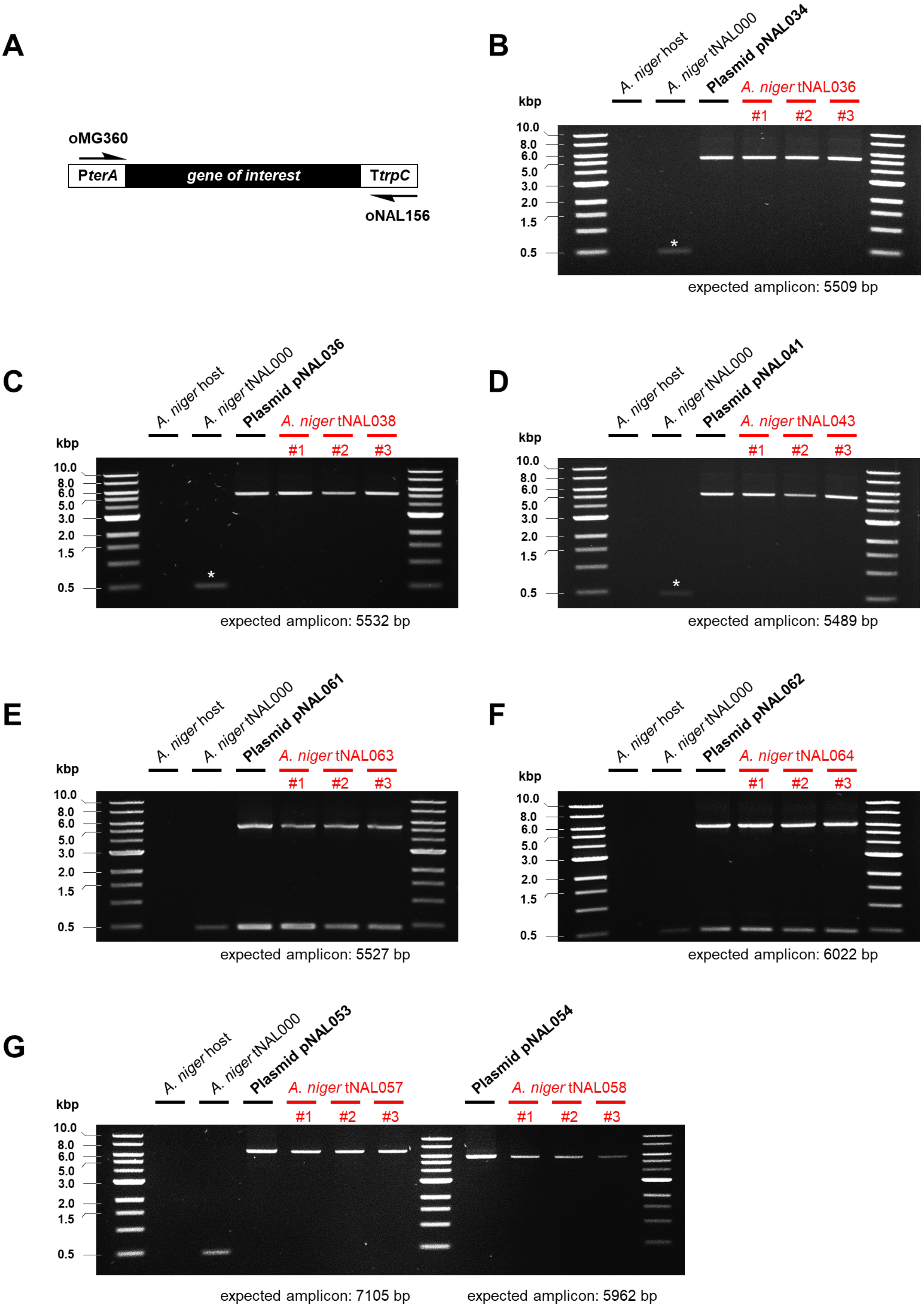
A
oMG360
PterA
gene of interest
TtrpC
oNAL156
B
A. niger host
A. niger tNAL000
Plasmid pNAL034
A. niger tNAL036
#1
#2
#3
kbp
10.0
8.0
6.0
5.0
3.0
2.0
1.5
0.5
*
expected amplicon: 5509 bp
C
A. niger host
A. niger tNAL000
Plasmid pNAL036
A. niger tNAL038
#1
#2
#3
expected amplicon: 5532 bp
D
A. niger host
A. niger tNAL000
Plasmid pNAL041
A. niger tNAL043
#1
#2
#3
expected amplicon: 5489 bp
E
A. niger host
A. niger tNAL000
Plasmid pNAL061
A. niger tNAL063
#1
#2
#3
expected amplicon: 5527 bp
F
A. niger host
A. niger tNAL000
Plasmid pNAL062
A. niger tNAL064
#1
#2
#3
expected amplicon: 6022 bp
G
A. niger host
A. niger tNAL000
Plasmid pNAL053
A. niger tNAL057
#1
#2
#3
Plasmid pNAL054
A. niger tNAL058
#1
#2
#3
expected amplicon: 7105 bp
expected amplicon: 5962 bp

Fig. S1 Agarose gel electrophoresis to verify integration of genes for basidiomycete PKSs in the genome of *A. niger* ATNT16Δ*pyrG*x24. **A** PCR strategy with oligonucleotide positions relative to the gene to verify the integrated expression cassette. *A. niger* host: DNA of the untransformed host strain *A. niger* ATNT16Δ*pyrG*x24 [1] was added for negative control; *A. niger* tNAL000 [2]: DNA of the *A. niger* host, transformed with insertless expression vector was added for negative control (highlighted with a white asterisk in case of a weak signal). For positive control, the PCR product obtained with the respective pNAL expression plasmid as template DNA is shown. Three independent transformants are shown per gene. **B** tNAL036: *A. niger* harboring the genes for CrPKS1. Expected amplicon: 5509 bp. **C** tNAL038: *A. niger* harboring the genes for CrPKS2. Expected amplicon: 5532 bp. **D** tNAL043: *A. niger* harboring the genes for CrPKS3. Expected amplicon: 5489 bp. **E** tNAL063: *A. niger* harboring the genes for PKS1ΔSAT. Expected amplicon: 5527 bp. **F** tNAL064: *A. niger* harboring the genes for PKS2ΔSAT. Expected amplicon: 6022 bp. **G** tNAL057 and tNAL058: *A. niger* harboring the genes for ArmB and ArmBΔSAT. Expected amplicons: 7105 bp and 5962 bp, respectively.

References

1. Geib E, Brock M. ATNT: an enhanced system for expression of polycistronic secondary metabolite gene clusters in *Aspergillus niger*. Fungal Biol Biotechnol. 2017;4:13.
2. Löhr NA, Eisen F, Thiele W, Platz L, Motter J, Hüttel W, Gressler M, Müller M, Hoffmeister D. Unprecedented mushroom polyketide synthases produce the universal anthraquinone precursor. Angew Chem Int Ed. 2022;61(24):e202116142.

A

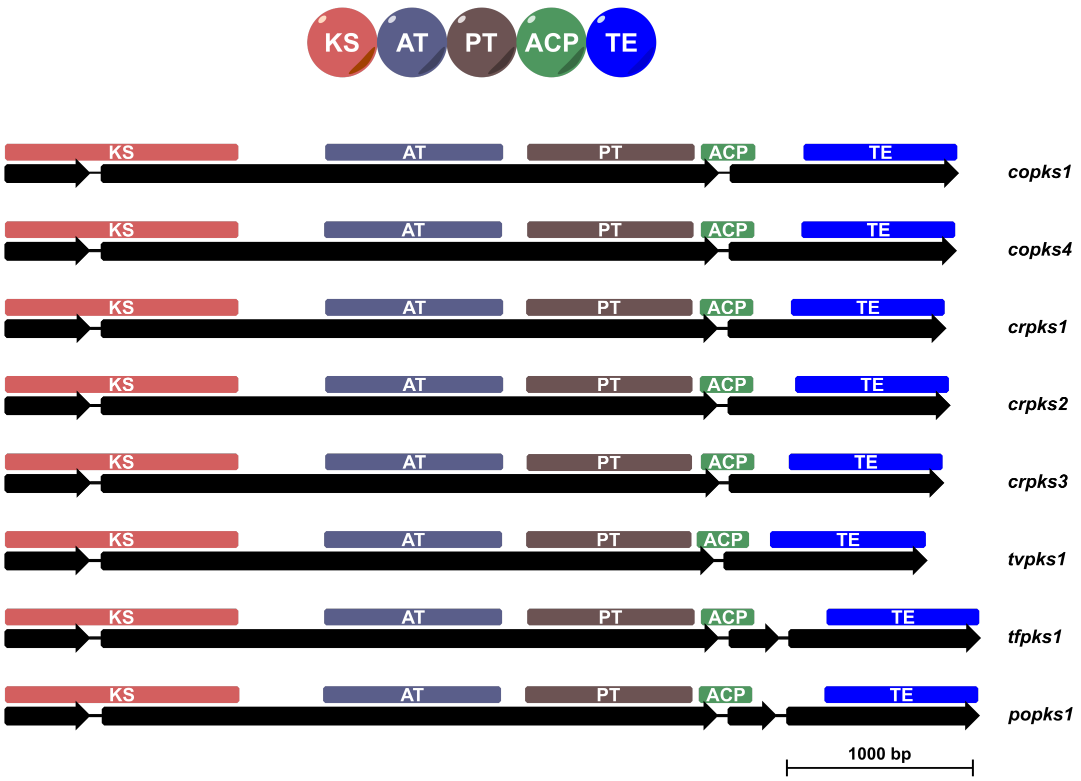

B

Genes	copks1	copks4	crpks2	crpks3	crpks1	tvpks1	tfpks1
copks4	88 %						
crpks1	83 %	83 %					
crpks2	84 %	84 %	92 %				
crpks3	83 %	83 %	87 %	88 %			
tvpks1	77 %	76 %	76 %	76 %	76 %		
tfpks1	84 %	83 %	82 %	82 %	81 %	78 %	
popks1	79 %	79 %	77 %	78 %	77 %	77 %	86 %

C

Enzymes	CoPKS1	CoPKS4	CrPKS1	CrPKS2	CrPKS3	TvPKS1	TfPKS1
CoPKS4	88 %						
CrPKS1	83 %	84 %					
CrPKS2	83 %	84 %	95 %				
CrPKS3	82 %	83 %	88 %	89 %			
TvPKS1	74 %	74 %	73 %	72 %	73 %		
TfPKS1	81 %	80 %	79 %	79 %	78 %	76 %	
PoPKS1	78 %	77 %	76 %	76 %	76 %	76 %	84 %

Fig. S2 Analyses of basidiomycete clade XII non-reducing polyketide synthases. **A** Domain architecture of proteins with their corresponding predicted gene structures. Domain acronyms are: KS: ketosynthase; AT: acyl transferase; PT: product template; ACP: acyl carrier protein; TE: thioesterase. **B** Heat map for pairwise percent sequence identities of the respective *pks* genes. **C** Pairwise percent sequence identity for the respective PKS enzymes.

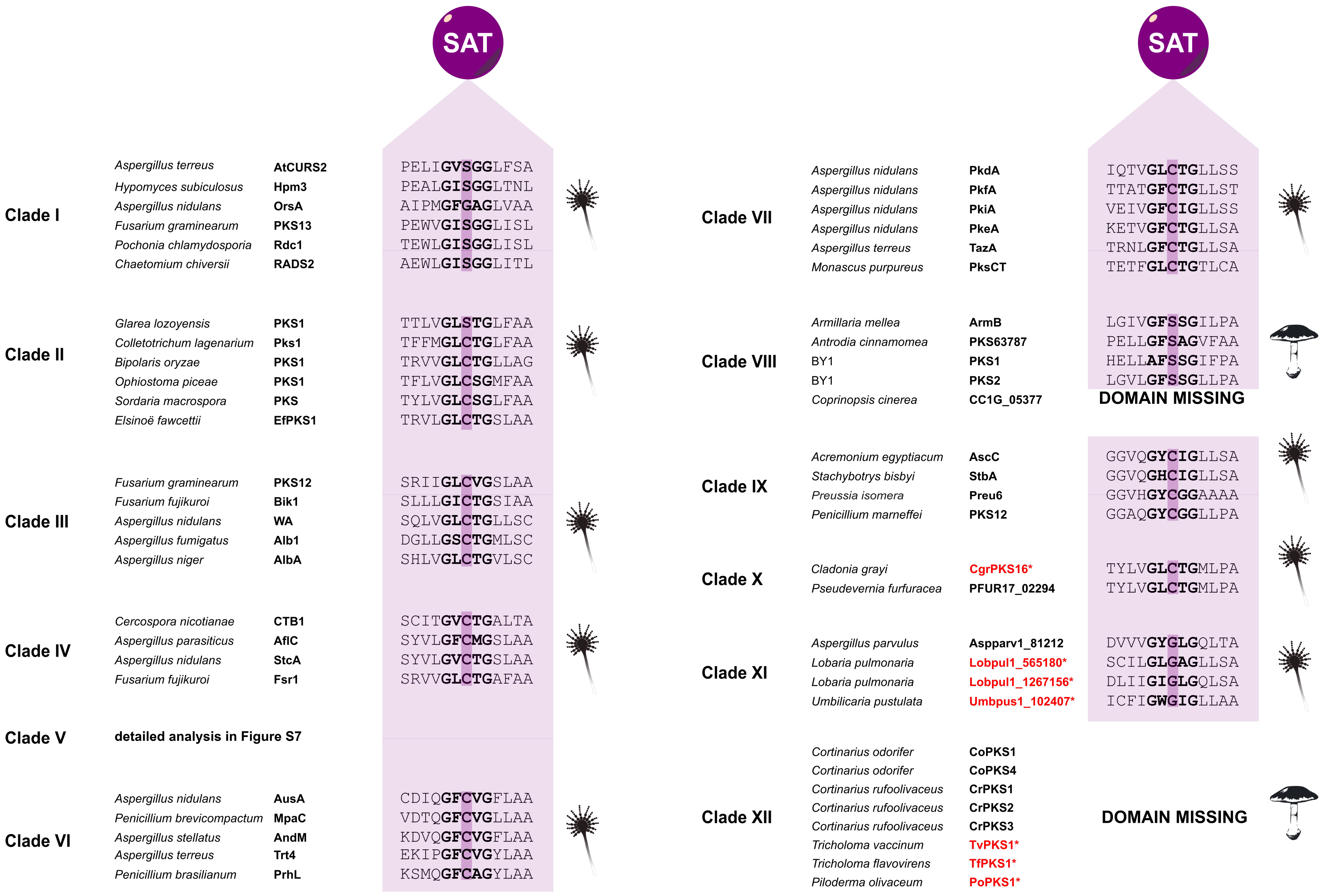

Fig. S3 Sequence analyses of the starter unit:acyl transferase (SAT) domains of fungal non-reducing polyketide synthases (NR-PKSs). The canonical catalytic motif Gx**C**xG/Gx**S**xG or the disrupted motif Gx**G**xG is highlighted in bold. Literature references for NR-PKSs are given in Table S7. Enzymes highlighted in red are either partially characterized or deduced based on sequence similarity.

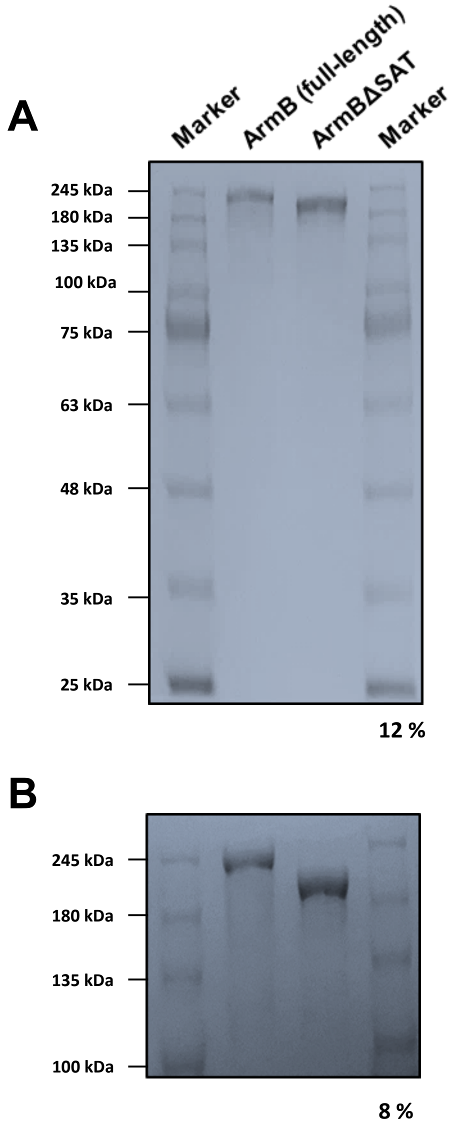

Fig. S4 SDS-polyacrylamide gel electrophoresis of ArmB and ArmBΔSAT. **A** Analysis on a 12% gel to verify the purity of the respective proteins. **B** Analysis on an 8% gel to visualize the mass shift between ArmB and ArmΔSAT. Left and right lanes: molecular weight marker. Center left lane: ArmB (full-length), recombinantly produced in *A. niger* tNAL057. Center right lane: ArmBΔSAT, recombinantly produced in *A. niger* tNAL058. Calculated masses are 239 kDa for ArmB (full-length) and 199 kDa for ArmBΔSAT.

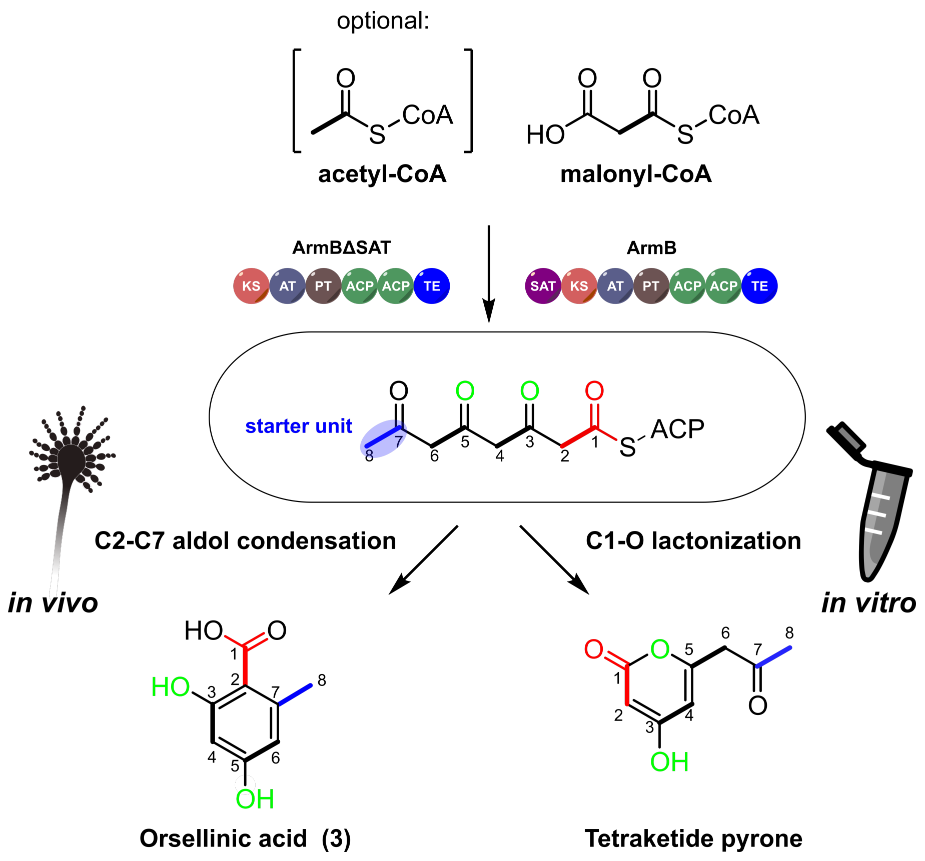

Fig. S5 Proposed cyclization patterns of the linear tetraketide to either orsellinic acid or tetraketide pyrone. Both enzymes, full-length ArmB and the truncated ArmBΔSAT produced orsellinic acid (**3**) *in vivo* in the heterologous host *Aspergillus niger* (Fig. 4) and the tetraketide pyrone *in vitro* (Fig. 5). While **3** is formed by C2-C7 aldol condensation, the pyrone can spontaneously arise from the linear tetraketide via C1-O-lactonization [1]. The incorporation of acetyl-CoA is optional, which led to the notion of a head start effect for SAT domain-independent synthesis of polyketides in mushrooms. Acetate units are highlighted in bold.

References

1. Feng Y, Yang X, Ji H, Deng Z, Lin S, Zheng J. The *Streptomyces viridochromogenes* product template domain represents an evolutionary intermediate between dehydratase and aldol cyclase of type I polyketide synthases. Commun Biol. 2022;5(1):508.

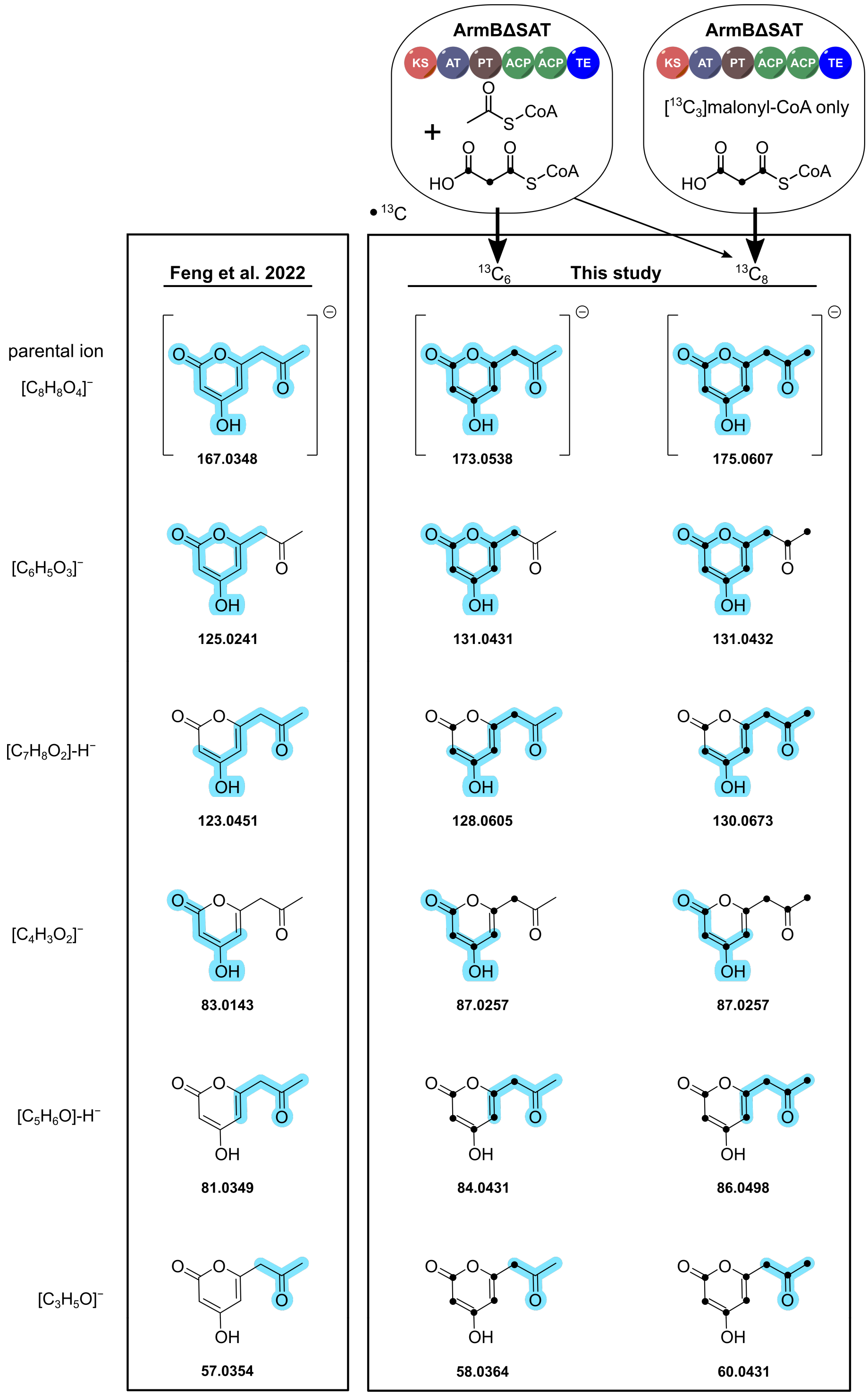

Fig. S6 Comparison of ESI-MS/MS fragmentation patterns of the tetraketide pyrone. The left panel shows the high-resolution ESI-MS/MS fragmentation pattern of the tetraketide pyrone, as published by Feng et al [1]. The right panel shows the masses detected in this study as a result of stable-isotope incorporation. Masses displayed refer to the assays of ArmBΔSAT with unlabeled acetyl-CoA and [$^{13}C_3$]malonyl-CoA (Fig. 6B) or with [$^{13}C_3$]malonyl-CoA (Fig. 6D) only. Structural proposals for fragments are highlighted in light blue and ^{13}C isotopes are marked with a bold dot.

References

1. Feng Y, Yang X, Ji H, Deng Z, Lin S, Zheng J. The *Streptomyces viridochromogenes* product template domain represents an evolutionary intermediate between dehydratase and aldol cyclase of type I polyketide synthases. Commun Biol. 2022;5(1):508.

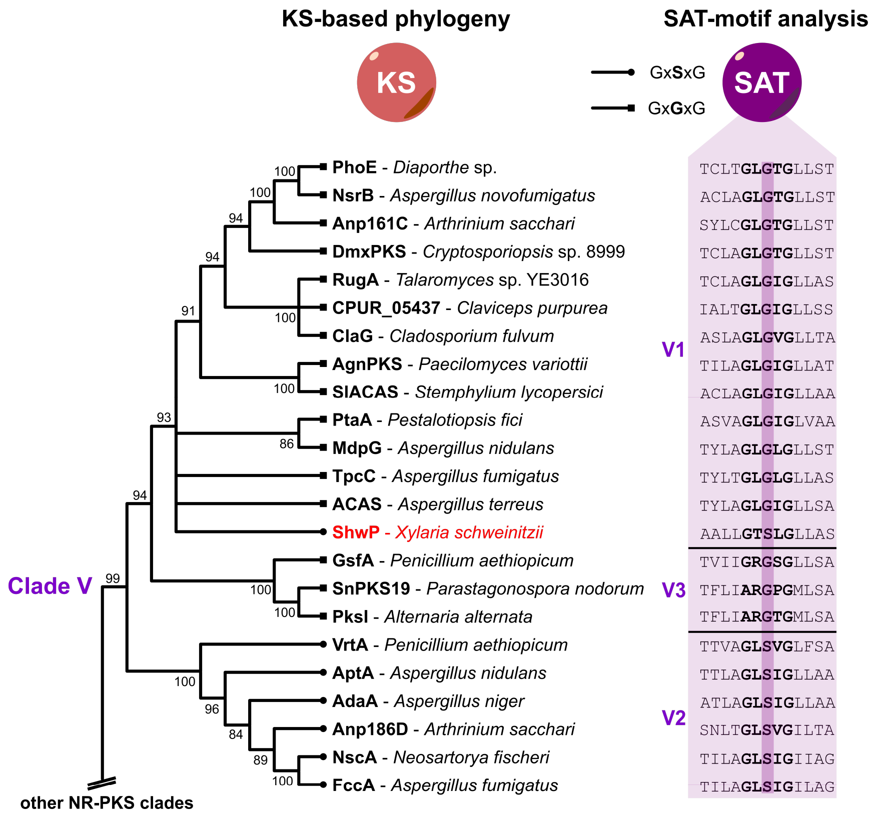

Fig. S7 Phylogenetic reconstitution of clade V non-reducing polyketide synthases (NR-PKSs) and sequence analyses of the respective starter unit:acyl transferase (SAT) domains. Shown is a magnified section of the maximum likelihood phylogenetic tree of fungal NR-PKSs (Fig. 2). The phylogeny was based on the ketosynthase domain (KS) of biochemically characterized NR-PKSs (listed in Table S7). Ultrafast bootstrap support values ≥75 are shown. The catalytic motif of the SAT domain [1] is highlighted in bold. An earlier phylogenetic classification divided clade V NR-PKSs into three subclades [2]. Our analysis revealed that enzymes of subclade V2 feature the canonical Gx**S**xG motif in the SAT domain, whereas members of the other clades (except ShwP, which has not yet been biochemically characterized (highlighted in red) [3]) possess a non-functional motif (Gx**G**xG). All subclades are well supported by bootstrap values > 90.

References

1. Crawford JM, Dancy BCR, Hill EA, Udwary DW, Townsend CA. Identification of a starter unit acyl-carrier protein transacylase domain in an iterative type I polyketide synthase. Proc Natl Acad Sci USA. 2006;103(45):16728-16733.
2. Throckmorton K, Wiemann P, Keller NP. Evolution of chemical diversity in a group of non-reduced polyketide gene clusters: using phylogenetics to inform the search for novel fungal natural products. Toxins. 2015;7(9):3572-3607.
3. Thiele W, Froede R, Steglich W, Müller M. Enzymatic formation of rufoschweinitzin, a binaphthalene from the basidiomycete *Cortinarius rufoolivaceus*. ChemBioChem. 2020;21(10):1423-1427.

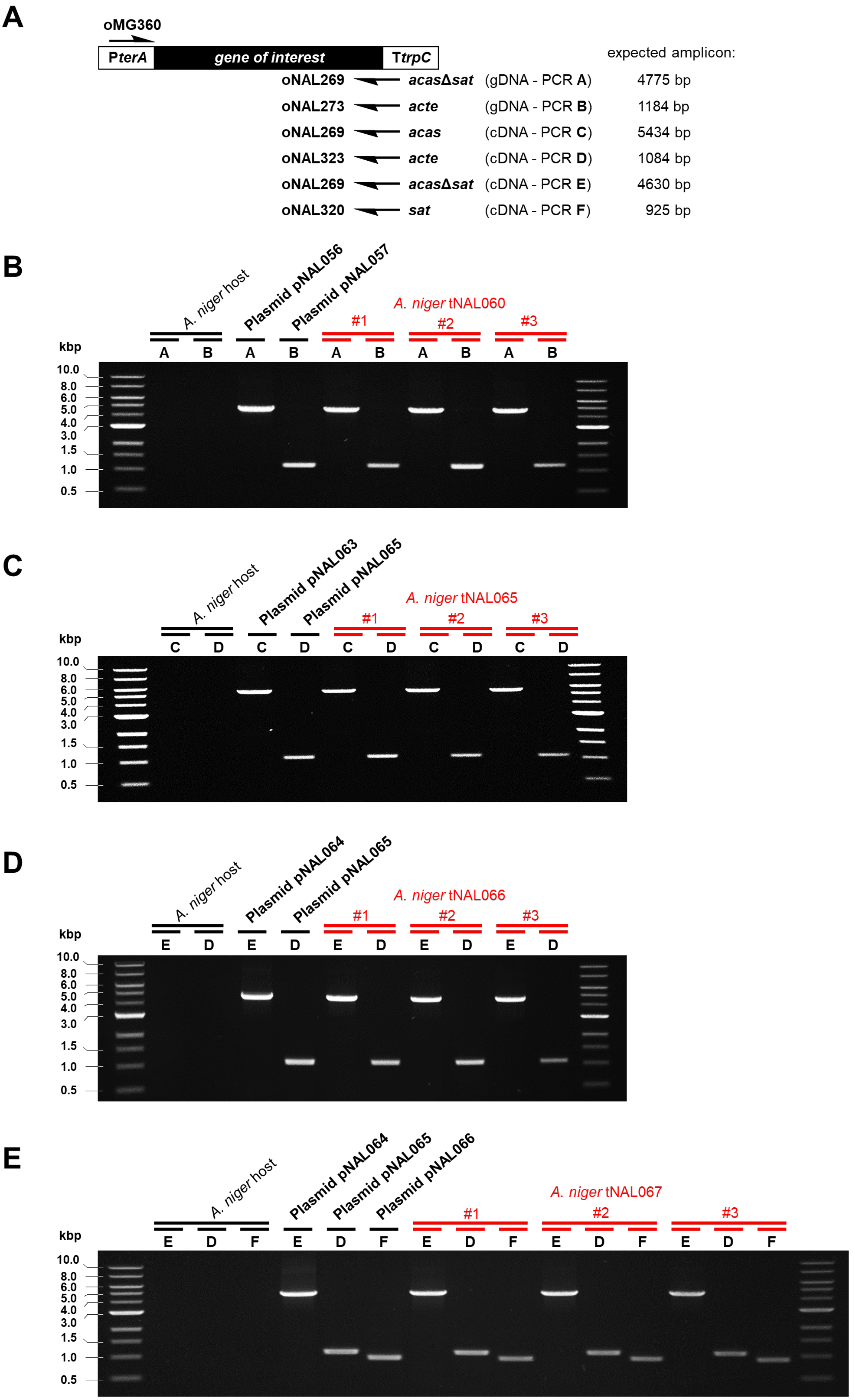
A
oMG360
PterA
gene of interest
TtrpC
expected amplicon:
oNAL269 acasΔsat (gDNA - PCR A) 4775 bp
oNAL273 acte (gDNA - PCR B) 1184 bp
oNAL269 acas (cDNA - PCR C) 5434 bp
oNAL323 acte (cDNA - PCR D) 1084 bp
oNAL269 acasΔsat (cDNA - PCR E) 4630 bp
oNAL320 sat (cDNA - PCR F) 925 bp
B
A. niger host
Plasmid pNAL056
Plasmid pNAL057
A. niger tNAL060
#1
#2
#3
kbp
A B A B A B A B A B
10.0
8.0
6.0
5.0
4.0
3.0
1.5
1.0
0.5
C
A. niger host
Plasmid pNAL063
Plasmid pNAL065
A. niger tNAL065
#1
#2
#3
kbp
C D C D C D C D C D
10.0
8.0
6.0
5.0
4.0
3.0
1.5
1.0
0.5
D
A. niger host
Plasmid pNAL064
Plasmid pNAL065
A. niger tNAL066
#1
#2
#3
kbp
E D E D E D E D E D
10.0
8.0
6.0
5.0
4.0
3.0
1.5
1.0
0.5
E
A. niger host
Plasmid pNAL064
Plasmid pNAL065
Plasmid pNAL066
A. niger tNAL067
#1
#2
#3
kbp
E D F E D F E D F E D F E D F
10.0
8.0
6.0
5.0
4.0
3.0
1.5
1.0
0.5

Fig. S8 Agarose gel electrophoresis to verify the integration of *acas* and *acte* genes in the genome of *A. niger* ATNT16_2_No. 17.1. **A** Oligonucleotide positions relative to the gene. In addition to oMG360, the specific oligonucleotide primers for Gibson assembly (Table S2) at the junction of the respective genes of interest and the *trpC* terminator were used. Expected amplicon lengths in case of an accurate genomic integration are shown. **B** tNAL060: *A. niger* harboring the genes (gDNA) for ACASΔSAT and ACTE. *A. niger* host: DNA of the untransformed host (ATNT16_2_No. 17.1) was added for negative control. For positive controls, the PCR products amplified from the respective expression plasmids (pNAL056 + pNAL057) as template DNA are shown. **C** tNAL065: *A. niger* harboring the genes (cDNA) for native full-length ACAS and ACTE. *A. niger* host: DNA of the untransformed host (ATNT16_2_No. 17.1) was added for negative control. For positive controls, the PCR products amplified from the respective expression plasmids (pNAL063 + pNAL065) as template DNA are shown. **D** tNAL066: *A. niger* harboring the genes (cDNA) for ACASΔSAT and ACTE. *A. niger* host: DNA of the untransformed host (ATNT16_2_No. 17.1) was added for negative control. For positive controls, the PCR products amplified from the respective expression plasmids (pNAL064 + pNAL065) as template DNA are shown. **E** tNAL067: *A. niger* harboring the genes (cDNA) for the discrete SAT domain of ACAS, the ACASΔSAT tetradomain and ACTE. *A. niger* host: DNA of the untransformed host (ATNT16_2_No. 17.1) was added for negative control. For positive controls, the PCR products amplified from the respective expression plasmids (pNAL064 + pNAL065 + pNAL066) as template DNA are shown.

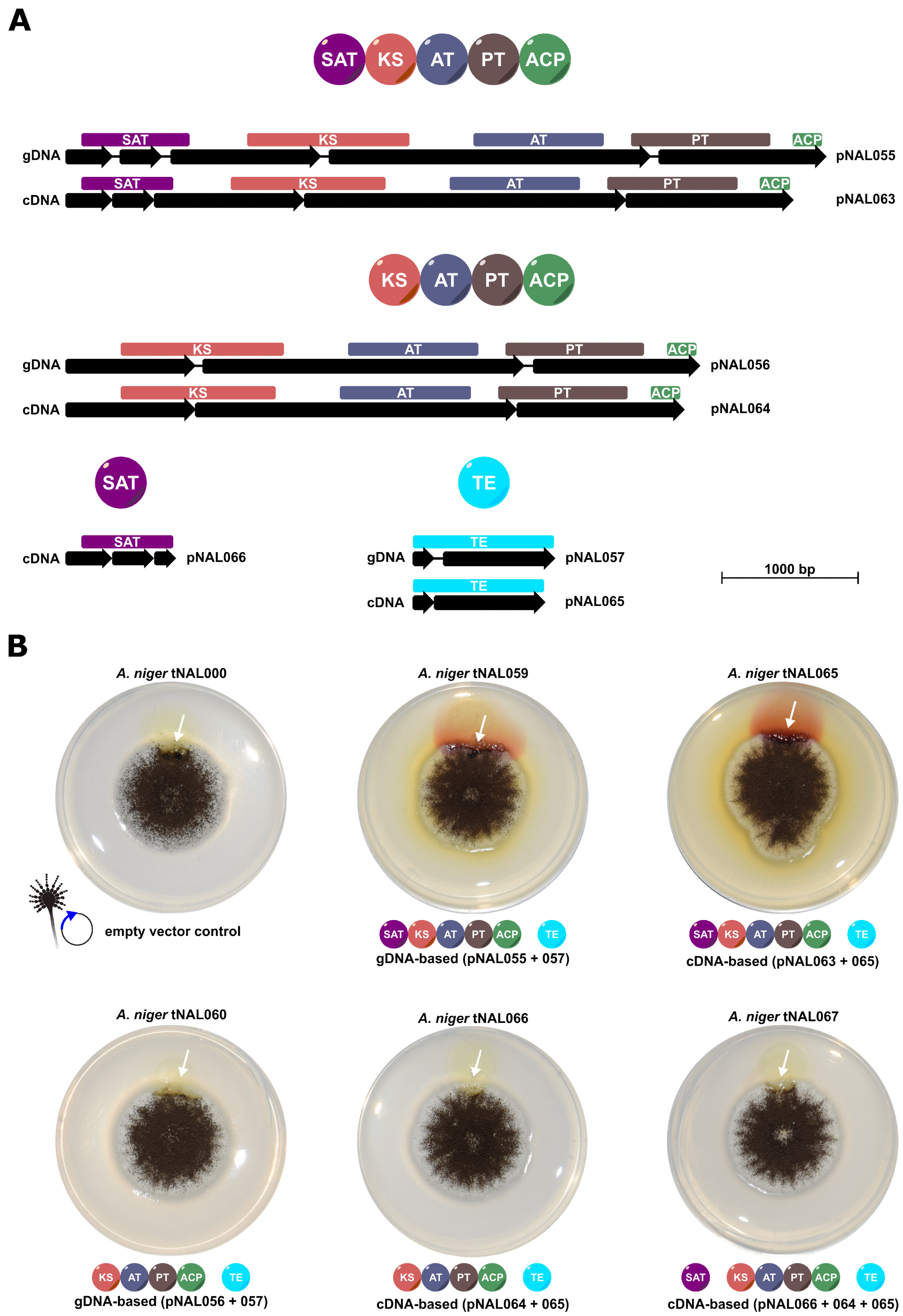

Fig. S9 Analyses of ACAS and ACASΔSAT in *A. niger* ATNT. **A** Domain architecture of complete or truncated ACAS [1] proteins with their corresponding gene structures. **B** *A. niger* transformants cultured under inducing conditions. A yellow area around *A. niger* tNAL059 [2] and tNAL065 indicated polyketide production. Application of 3 N NaOH (white arrow) turned this region reddish. LC-MS analyses of the transformants are shown in Fig. S10.

References

1. Awakawa T, Yokota K, Funa N, Doi F, Mori N, Watanabe H, Horinouchi S. Physically discrete beta-lactamase-type thioesterase catalyzes product release in atrochrysone synthesis by iterative type I polyketide synthase. Chem Biol. 2009;16(6):613-623.
2. Löhr NA, Urban MC, Eisen F, Platz L, Hüttel W, Gressler M, Müller M, Hoffmeister D. The ketosynthase domain controls chain length in mushroom oligocyclic polyketide synthases. ChemBioChem. 2023;24(3):e202200649.

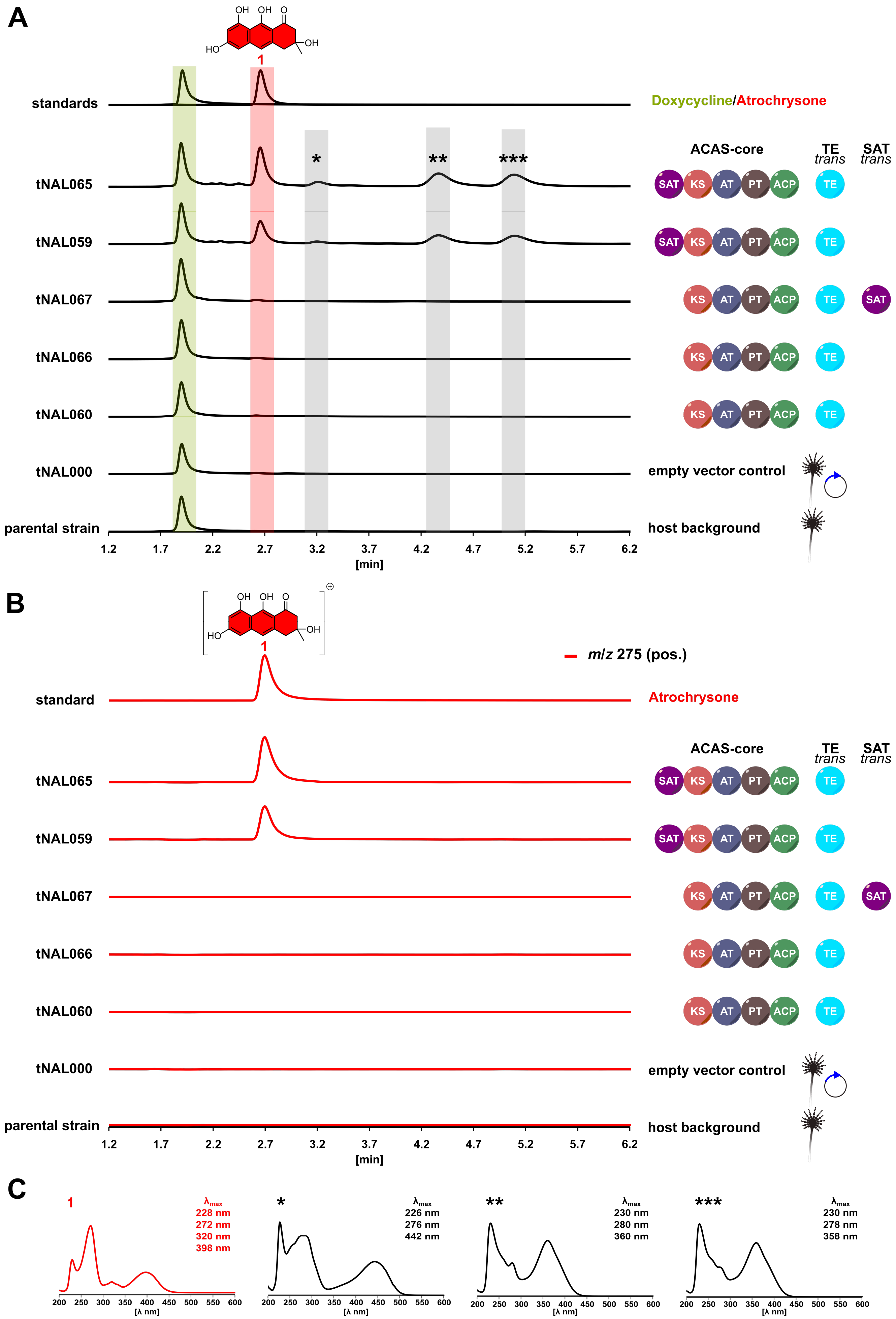

A
OH OH O
HO
OH
1
standards
tNAL065
tNAL059
tNAL067
tNAL066
tNAL060
tNAL000
parental strain
*
**
***
1.2 1.7 2.2 2.7 3.2 3.7 4.2 4.7 5.2 5.7 6.2
[min]
Doxycycline/Atrochrysone
ACAS-core
TE trans
SAT trans
SAT KS AT PT ACP TE
KS AT PT ACP TE SAT
empty vector control
host background
B
m/z 275 (pos.)
standard
Atrochrysone
C
1
λmax
228 nm
272 nm
320 nm
398 nm
*
226 nm
276 nm
442 nm
**
230 nm
280 nm
360 nm
***
230 nm
278 nm
358 nm
200 250 300 350 400 450 500 550 600
[λ nm]

Fig. S10 *In vivo* activity assays of ACAS and ACASΔSAT variants in *Aspergillus niger*. **A** Chromatograms were recorded at λ = 395 nm. The top lane represents an overlay of authentic standards of atrochrysone (**1**) and doxycycline. The latter was added to induce gene expression in *A. niger* ATNT16_2_No. 17.1. Lower lanes: *A. niger* expressing the genes for full-length ACAS and ACTE of *A. terreus* [1] (based on cDNA; in *A. niger* tNAL065), full-length ACAS and ACTE (based on gDNA; in *A. niger* tNAL059 [2]), ACASΔSAT, ACTE along with the discrete SAT monodomain of ACAS (based on cDNA; tNAL067), ACASΔSAT and ACTE (based on cDNA; tNAL066) and ACASΔSAT and ACTE (based on gDNA, tNAL060). *A. niger* tNAL000 [3], containing the insertless expression vector phis_SM-Xpress_URA, and the parental strain *A. niger* ATNT16_2_No. 17.1 were included as negative controls to monitor the metabolic background of the host. In addition to **1**, we also detected the common set of minor follow-up compounds that are concomitantly formed upon heterologous production of ACAS-type enzymes in aspergilli [1, 3-5], consisting of endocrocin (*) and its anthrone dimers (**/***). **B** Extracted ion chromatograms are shown for m/z 275 $[M+H]^+$ for optimum sensitivity to detect **1**. **C** UV/Vis spectra of **1**, endocrocin (*) and its anthrone dimers (**/***) of the transformant *A. niger* tNAL065. The local maxima are in agreement with literature data [3].

References

1. Awakawa T, Yokota K, Funa N, Doi F, Mori N, Watanabe H, Horinouchi S. Physically discrete beta-lactamase-type thioesterase catalyzes product release in atrochrysone synthesis by iterative type I polyketide synthase. Chem Biol. 2009;16(6):613-623.
2. Löhr NA, Urban MC, Eisen F, Platz L, Hüttel W, Gressler M, Müller M, Hoffmeister D. The ketosynthase domain controls chain length in mushroom oligocyclic polyketide synthases. ChemBioChem. 2023;24(3):e202200649.
3. Löhr NA, Eisen F, Thiele W, Platz L, Motter J, Hüttel W, Gressler M, Müller M, Hoffmeister D. Unprecedented mushroom polyketide synthases produce the universal anthraquinone precursor. Angew Chem Int Ed. 2022;61(24):e202116142.
4. Chiang YM, Szewczyk E, Davidson AD, Entwistle R, Keller NP, Wang CCC, Oakley BR. Characterization of the *Aspergillus nidulans* monodictyphenone gene cluster. Appl Environ Microb. 2010;76(7):2067-2074.
5. Griffiths S, Mesarich CH, Saccomanno B, Vaisberg A, De Wit PJGM, Cox R, Collemare J. Elucidation of cladofulvin biosynthesis reveals a cytochrome P450 monooxygenase required for anthraquinone dimerization. Proc Natl Acad Sci USA. 2016;113(25):6851-6856.
